# Programme Reporting Standards (PRS) for improving the reporting of sexual, reproductive, maternal, newborn, child and adolescent health programmes

**DOI:** 10.1186/s12874-017-0384-7

**Published:** 2017-08-03

**Authors:** Anna E. Kågesten, Özge Tunçalp, Anayda Portela, Moazzam Ali, Nhan Tran, A. Metin Gülmezoglu

**Affiliations:** 1 Department of Population, Family and Reproductive Health, Johns Hopkins School of Public Health, Baltimore, MD USA; 2 Department of Reproductive Health and Research, including the UNDP/UNFPA/UNICEF/WHO/World Bank Special Programme of Research, Development and Research Training in Human Reproduction, World Health Organization, Geneva, Switzerland; 3 Department of Maternal, Child and Adolescent Health, World Health Organization, Geneva, Switzerland; 4 Implementation Research Platform, Alliance for Health Policy and Systems Research, World Health Organization, Geneva, Switzerland

**Keywords:** Reporting, Guideline, Programme, Intervention, Implementation, Context, Sexual and Reproductive Health, Maternal Health, Public Health, Family planning, Safe abortion

## Abstract

**Background:**

Information about design, implementation, monitoring and evaluation is central to understand the impact of programmes within the field of sexual, reproductive, maternal, newborn, child and adolescent health (SRMNCAH). Existing reporting guidelines do not orient on reporting of contextual and implementation issues in sufficient detail. We therefore developed Programme Reporting Standards (PRS) to be used by SRMNCAH programme implementers and researchers.

**Methods:**

Building on the first step of the PRS development (a systematic review to identify reporting items), we conducted a three-round online Delphi consensus survey with experts. Consensus was defined a-priori as 80% agreement of items as essential. This was followed by a technical consultation with a group of experts to refine the items, definitions and their structuring. The revised PRS was piloted to assess its relevance to current SRMNCAH programme reports and identify key issues regarding the use of the PRS.

**Results:**

Of the 81 participants invited to the Delphi survey, 20 responded to all three rounds. In the final round, 27 items received consensus as essential; three items were ranked as “borderline” essential; 20 items as supplementary. The items were subsequently revised, followed by a technical consultation with 29 experts to further review and refine the PRS. The feedback resulted in substantial changes to the structure and content of the PRS into 24 items across five domains: *Programme overview; Programme components and implementation; Monitoring of Implementation*; *Evaluation and Results*; and *Synthesis.* This version was used in a piloting exercise, where questions regarding how much information to report and how to comment on the quality of the information reported were addressed. All items were kept in the PRS following the pilot although minor changes were made to the flow and description of items.

**Conclusions:**

The PRS 1.0 is the result of a structured, collaborative process, including methods to incorporate input from SRMNCAH stakeholders. The World Health Organization will develop a document that explains the items in greater detail, and will also apply the PRS to on-going initiatives. We welcome continuous input from the field, while it is being used, to improve its relevance and usefulness.

**Electronic supplementary material:**

The online version of this article (doi:10.1186/s12874-017-0384-7) contains supplementary material, which is available to authorized users.

## Background

There is evidence that high-quality, evidence-based programmes within the field of sexual, reproductive, maternal, child and adolescent health (SRMNCAH) can lead to substantial improvements in health outcomes. As highlighted in the Every Women Every Child *Global Strategy for Women’s, Children’s and Adolescents’ health (*2016–2030), we have more knowledge than ever before to end preventable deaths and improve the health and wellbeing of individuals across the life course through the use of evidence-based interventions [1].

In order to sustain the current progress and support the further implementation and scaling-up of identified evidence-based SRMNCAH interventions, programmes need to understand not only if an intervention worked (or not), but also how, why and for whom it was successful, as well as information regarding the context in which the intervention was taken forward [2, 3]. This requires adequate and transparent documentation about how programmes were designed, implemented, monitored and evaluated. However, the complex realities of many programmes including their implementation make it difficult to communicate such processes to an external audience. Indeed, the successful implementation and impact of many programmes - particularly those of social and behavioural nature - is very much tied to the “real world” context (e.g. sociocultural, socioeconomic, geographical, legal, political, health system), which may not be easily described [3, 4]. Insufficient detail on the context and implementation does not only hamper replication and scale-up, but contributes to the gap between research and practice [5]. A standardized way of reporting on the processes and contextual elements of programmes throughout the different phases of a programme cycle would allow for conceptualization of information needs from the start of a programme, and also facilitate better documentation and synthesis of elements critical to implementation and sustainability.

Over the last few years, a number of guidelines have been developed to improve the (often inadequate [6, 7]) description of interventions in research articles. For example, the *Template for Intervention Description and Replication* (TIDieR) provides a 12-items checklist for describing clinical interventions in “sufficient detail to allow replication” [8]. In addition, the on-going development of the *UNTIDieR* standards aims to provide a framework for the reporting of public health and social policy interventions [9]. Other relevant guidelines include the *Standards for Reporting Implementation Studies of Complex Interventions* (StaRI) [10], and the *Reporting Guidelines for Implementation and Operational Research* endorsed by the Bulletin of the World Health Organization to be used by authors of implementation science articles [2]. In addition to the existing standards for different research study designs (see Additional file 1), these guidelines all have in common that they strive to reduce the gap between what is learnt in the field and what is communicated through scientific publications. However, while relevant, they were mostly developed for research reporting and do not cover all relevant aspects of programme design, development, implementation and evaluation processes and outcomes. In addition, the traditional structure of a scientific research article may not be the most appropriate space for many programmes to report on contextual issues or to elaborate on the implementation of components in sufficient detail to allow others to learn from their experiences.

To address this gap and complement existing reporting frameworks, we developed Programme Reporting Standards (PRS) to be used by programme implementers and researchers in the field of SRMNCAH. The overarching goal of the PRS is to provide guidance for complete and accurate reporting on the design, implementation, monitoring and evaluation processes of SRMNCAH programmes. The initiative is a collaborative effort led by the World Health Organization (WHO) Department of Reproductive Health and Research, including the UNDP/UNFPA/UNICEF/WHO/World Bank Special Programme of Research, Development and Research Training in Human Reproduction (HRP), and the Department of Maternal, Newborn, Child and Adolescent Health, in partnership with the Alliance for Health Policy and Systems Research hosted by the WHO.

### Overview of PRS development

We developed the PRS in four steps in line with recommendations for the development of health research reporting guidelines [11]: 1) systematic review, 2) three-round Delphi exercise, 3) face-to-face consensus meeting, and 4) piloting through existing programmes.

In the first step we conducted a systematic review of existing reporting guidelines, checklists and other tools, applicable for reporting on programmes targeting sexual and reproductive health (SRH) outcomes. We identified a total of 50 reporting items (Additional file 2), which formed the basis for the subsequent steps of the PRS development. A detailed description of the review, including the methods (screening, data extraction and synthesis) and results have been described elsewhere [12]. While we initially set out to develop the PRS specific to SRH programmes, the systematic review captured reporting tools related to diverse health programmes. In fact, all of the identified items were applicable to programmes within the broader frame of SRMNCAH, and we therefore decided to expand the PRS to these areas.

The subsequent phases of development aimed to determine consensus among experts about which items should be included in a PRS through an initial Delphi exercise (step 2) followed by a technical consultation (step 3), and then a pilot-test of the PRS in order to assess its feasibility and relevance in relation to existing programmes in the field of SRMNCAH (step 4). In this paper, we present results from these steps and introduce the PRS version 1.0. We also summarize the next steps to ensure its uptake and use by programmes.

## Methods

### Delphi exercise to reach consensus on core PRS items

We undertook a three-round Delphi survey with experts in the field of SRMNCAH to refine and revise the list of 50 items generated in the systematic review [12] by identifying those of highest relevance for the PRS. The Delphi survey technique (Delphi for short) is a structured method to explore, seek consensus and corroborate judgements on a specific topic [13]. Originally developed by the RAND Corporation in the 1950s, the method seeks the opinion of experts through an iterative series of structured survey rounds. Throughout the process, the responses and feedback from participants is fed into the next round until consensus has been reached [14].

### Delphi participants and procedure

We invited 81 experts in the areas of implementation, research and funding of SRMNCAH programmes and/or reporting guideline development to participate in an iterative Delphi survey. Using a literature review and global WHO networks, participants were selected because of their specific expertise within these areas, with the goal of having different organizational perspectives (e.g. non-governmental, governmental, donor, universities, UN-bodies) and geographical representation. The Delphi process consisted of three prospectively planned rounds, with the first round starting in September 2015 and the last round concluding in March 2016. Participants had about two to four weeks to respond to each survey round, and reminders were sent after two and three weeks, respectively. Participants were initially encouraged to complete all survey rounds, however invitations for the second and third rounds were restricted to those that responded to the previous round to ensure continuity in responses. For each round, participants received personalized emails containing a link to an on-line survey administered through the electronic instrument *SurveyMonkey* (www.surveymonkey.com).

### Rounds 1 and 2

The purpose of the first scoring round was to introduce participants to the list of items generated from the systematic review, produce initial rankings of the relevance of each item for a PRS, and obtain suggestions for item revisions and their descriptions. Participants were asked to rate the relevance of the items identified in the systematic review using a Likert-type scale ranging from 1 (not important) to 9 (essentially important), and encouraged to suggest new items as well as modification to the structure and language of items.

Participants who completed round 1 were sent a summary of the results (ranking of each items and a revised list of items) and invited to rank the importance of the revised items using the same 9-point scale. Participants were also invited to comment on the structure or language of items and their descriptions.

*Quantitative analysis of items scorings:* For rounds 1 and 2, we calculated descriptive statistics including mean (SD), median (IQR), minimum and maximum scores for each item and across items. We allocated the scores of each item into three categories of importance: *not important* (mean scores 1–3); *important or desirable but not essential* (mean scores 4–6); and *essentially important* (mean scores 7–9). These categories were used to estimate the percent agreement between participants, defined as the proportion of participants rating an item in the same category of importance. A-priori level of consensus was set to 80% agreement of items as essentially important (mean scores ≥7 on a 1–9 scale).

*Qualitative analysis of open-ended comments:* We used thematic analysis to synthesize open-ended comments for rounds 1 and 2. Following open-ended coding of relevant text units of each comment, item suggestions were categorized into the PRS domains and sub-domains identified in the systematic review. New sub-domains were created when applicable. For each new item, we summarized the number of participants suggesting the item, provided an explanation of the item, and one or more examples of the participant’s original comment (verbatim). Additional items had to be suggested by at least two participants in order to be included in the subsequent survey round. Comments on the structure or wording of items were reviewed and clustered into similar categories or themes.

### Round 3

The goal of the third and final Delphi round was to reach consensus on items to be included in the PRS as essential or supplementary. Scoring results were once more sent back to participants who completed round 2 together with an updated list of items. Based on the rankings from round 2 (during which no items were ranked as “not important”, as further described in the results), we assigned all items into one of two categories, and asked participants to indicate their agreement with this categorization of items as: *essential* (include in a PRS tool, ranked as essential by at least 80% of participants in round 2) or *supplementary* (received lower consensus, but were still ranked as important in round 2).

Responses from round 3 were used to estimate the percent agreement between participants as the proportion of participants rating an item in the same category of importance (e.g. the % consistently ranking an item as essentially important in rounds 2 and 3).

### Technical consultation to refine and finalize draft PRS

In the next step of the PRS development, we convened a technical face-to-face consultation in July 2016 at the WHO headquarters in Geneva to review the results of the systematic review and the Delphi survey in order to further refine and develop the next version of the PRS. Twenty-nine experts attended the meeting; of these, three had participated in the Delphi exercise, six were staff members or consultants at the WHO, and 20 had not previously participated. While we invited all Delphi participants who completed the three rounds, most responded that they were unable to take part in the consultation due to prior commitments. Additional experts (*n* = 20) were therefore identified to ensure coverage of the different health areas of SRMNCAH, expertise in different types of health programmes (e.g. service delivery, advocacy, social and behavioural interventions), and representation of different organizations. Plenary discussions and group work were organized to allow for a more in-depth review and discussion of the different items than the online-Delphi had allowed for, with specific focus on the organization, wording and relevance (essential vs. supplementary) of the items. Throughout the meeting, we strived to reach consensus on which items should be included in the PRS, and to identify concrete suggestions for how to improve the structure and flow of the PRS so as to make it user-friendlier.

After the meeting, a revised version was distributed to the meeting participants to ensure that the new version had adequately captured the agreements reached in the meeting. Their feedback was in turn used to produce a penultimate version, which served as the basis for the piloting described below.

### Piloting the draft PRS in existing programmes

In the final step of the PRS development, we pilot-tested the PRS during October and November 2016 to assess its relevance and fit to existing SRMNCAH programme reports, and to identify issues and questions regarding its use. In this phase the PRS was used to report on an already completed programmes. Partner organizations nominated programmes of which four were selected to represent different SRMNCAH topics and to create a balance in the programme type (e.g. service delivery, communication, prevention), duration and implementation scale. A programme staff or other representative with good knowledge of the programme activities were asked to gather reports and other forms of documentation relevant to understanding the development, implementation and evaluation processes, and to complete the PRS items by indicating the source and page numbers where the corresponding information could be located. The participants were also asked to provide their feedback related to the completeness, logical flow and formatting of the PRS, definitions and explanations, and other issues or questions. A second reviewer (AEK) verified the information so as to determine if the correct information was reported to the corresponding item. A virtual meeting was held with the team who participated in the pilot to discuss and clarify the feedback and determine final changes to be made. The results from the pilot exercise were used to update the PRS into the final version presented in this paper.

## Results

### Delphi results

#### Participant characteristics

Of the 81 experts initially invited, 59.3% (*N* = 48) responded to the first round of the Delphi exercise. Out of these, 66.7% (*N* = 32) completed the second survey round and 41.7% (*N* = 20) responded to all three rounds. Among those that completed all three rounds, 40% represented Universities and 25% non-governmental organisations (NGOs), and the remaining worked for donor agencies (15%), UN bodies (10%), governments (5%), or other organisations (5%). As was the case for previous rounds, most participants in round 3 reported a background in research/academia (55%) and/or programme planning/implementation (45%), followed by management (40%) and medical/clinical (35%) areas (multiple response options possible). Over two-thirds of participants in round 3 (70%) came from organizations that conduct global work, followed by regional representation from Africa and Southeast Asia (10% respectively), Western Pacific and North America (5% respectively) (Table 1).

**Table 1:** Characteristics of participants across Delphi rounds

	Round 1 (*N* = 48)		Round 2 (*N* = 29)		Round 3 (*N* = 20)	
	N	%	N	%	N	%
Organization						
UN	5	10.4%	3	10.7%	2	10%
University	16	33.3%	10	35.7%	8	40%
NGO	14	29.2%	8	28.6%	5	25%
Hospital/clinic	2	4.2%	0	0%	0	0%
Donor	4	8.3%	3	10.7%	3	15%
Government	5	10.4%	3	10.7%	1	5%
Other	2	4.2%	1	3.6%	1	5%
Professional background^a^						
Programme planner/implementer	25	52%	13	44.8%	9	45%
Researcher/academic	31	65.3%	15	51.7%	11	55%
Medical/clinical	16	32.7%	8	27.6%	7	35%
Management	19	37.8%	10	34.5%	8	40%
Other	6	12.2%	5	17.2%	2	10%
Region						
Global	33	68.8%	21	72.4%	14	70%
Africa	6	12.5%	3	10.3%	2	10%
Western pacific	2	4.2%	1	3.4%	1	5%
Southeast Asia	3	6.3%	2	6.9%	2	10%
North America	3	6.3%	2	6.9%	1	5%
Europe	1	2.1%	0	0%	0	0%

^a^Multiple response options possible

#### Item rankings and suggestions

Table 2 presents an overview of the scoring and changes made to items in rounds 1 and 2. Most items in the first round received high ratings with mean scores ranging from 8.8 (*Overall goal/objectives of programme*) to 6.2 (*Innovation*). No items were ranked as “not important” (received a mean score below 4). Out of the 50 items identified in the systematic review, 23 items received high consensus (ranked as essentially important by 80% or more of the participants). Based on the open-ended comments received, we made several revisions to the wording and description of items, merged and added new items, resulting in 50 items for the second scoring round.

**Table 2:** Summary of Delphi rounds 1–3 rankings of reporting items

Round 1 (*N* = 43)					Round 2 (*N* = 29)					Round 3 (*N* = 20)	
Original items (systematic review)	Rating scores	Category of importance (% of respondents)^a^			Revised items post round 1	Rating scores	Category of importance (% of respondents)^a^			Revised items post round 2	Item ranking^b^
	Mean (SD)	Ess.	Imp.	Notimp.		Mean (SD)	Ess.	Imp.	Notimp.		
*Programme Preparation*					*Programme Preparation*					*Programme Preparation*	
1. Programme name	6.9 (2.2)	58.1	37.2	4.7	1. Programme name	7.2 (1.7)	62.1	34.5	3.5	1. Programme name	Suppl.
2. Overall goal/objectives – anticipated impact	8.8 (0.6)	97.7	2.3	–	2. Objectives and anticipated effects	8.7 (0.7)	96.6	3.5	–	2. Objectives and anticipated effects	Ess.
3. Target population	8.4 (1.4)	93.0	4.7	2.3	3. Target population and area	8.5 (0.8)	96.6	3.5	–	3. Target population and area	Ess.^c^
4. Organization/agency	6.4 (2.0)	48.8	44.2	7	4. Partners and stakeholder involvement	7 (1.5)	65.5	34.5	–	4. Partners and stakeholder involvement	Suppl.
5. Funding source	6.2 (1.7)	39.5	58.1	2.3	5. Funding source	6.4 (1.7)	44.8	48.4	6.9	5. Funding source	Suppl.
6. Programme design process	7.2 (1.6)	69.8	27.9	2.3	6. Programme design process	7 (1.5)	62.1	37.9		6. Programme design process	Suppl.
7. Theoretical foundation	7.3 (1.8)	79.1	16.3	4.7	7. Theory and/or logic model	7.6 (1.4)	82.8	13.8	3.5	7. Theory and/or logic model	Ess.
8. Program manual	6.7 (1.6)	55.8	41.9	2.3	8. Program manual	5.8 (1.8)	41.4	48.3	10.3	8. Program manual	Suppl.
9. Implementation strategy	7.8 (1.4)	81.4	18.6	–	9. Implementation strategy	7.8 (1.2)	82.8	17.2	–	9. Implementation strategy	Ess.
10. Evaluation plans	8.4 (1.1)	93.0	7.0	–	10. Evaluation plans	7.9 (1.2)	85.7	14.3	–	10. Evaluation plans	Ess.
					11. Ethical considerations	7.4 (1.4)	72.4	27.6	–	11. Ethical considerations	Suppl.
					12. Dissemination plans	6.5 (1.9)	55.2	41.4	3.5	12. Dissemination plans	Suppl.
11. Piloting of activities		79.1	20.9	–	13. Piloting of activities	7 (1.2)	62.1	37.9	–	13. Piloting of activities	Suppl.
*Programme*				–	*Programme*				–	*Programme*	
*Implementation*					*Implementation*					*Implementation*	
12. Components/activities	8.4 (1.1)	88.4	11.6	–	14. Components/activities	8.3 (1.2)	89.7	10.3	–	14. Components/ activities	Ess.
13. Complexity	7.2 (1.4)	66.7	33.3	–	15. Complexity	6.8 (1.2)	37.9	62.1	–	*Merged with #14*	
14. Standardisation	7.5 (1.3)	81.4	18.6	–	16. Standardisation and tailoring	7.7 (1)	86.2	13.8	–	15. Standardisation and tailoring	Ess.
15. Innovation	6.2 (1.7)	44.2	48.8	7.0	Merged with #14						
16. Materials	7.2 (1.3)	72.1	27.9	–	17. Materials	6.8 (1.6)	58.6	37.9	3.5	16. Materials	Suppl.
17. Timing (when)	8.0 (1.4)	83.3	16.7	–	18. Timing (when)	7.9 (1.4)	79.3	20.7	–	17. Timing (when)	Ess.^c^
18. Setting (where)	8.5 (1.0)	92.9	7.1	–	19. Setting (where)	8.6 (0.8)	96.6	3.5	–	18. Setting (where)	Ess.
19. Dose and intensity (how much)	8.4 (1.1)	92.9	7.1	–	20. Dose and intensity (how much)	8 (1.2)	86.2	13.8	–	19. Dose and intensity (how much)	Ess.
20. Provider characteristics (by whom)	7.3 (1.5)	71.4	28.6	–	21. Provider/staff characteristics (by whom)	7.3 (1.4)	72.4	27.6	–	20. Provider/staff characteristics (by whom)	Suppl.
21. Provider/staff training	7.3 (1.6)	69.1	31	–	22. Provider/staff training	7.1 (1.7)	65.5	31.0	3.5	21. Provider/staff training	Suppl.
22. Provider reflexivity	6.4 (1.9)	54.8	35.7	9.5	23. Provider reflexivity	6.2 (1.4)	46.4	53.6	–	22. Provider reflexivity	Suppl.
23. Participant recruitment	7.8 (1.4)	78.6	21.4	–	24. Participant recruitment	7.5 (1.3)	75.0	25.0	–	23. Participant recruitment	Suppl.
24. Participants (who)	8.2 (1.1)	92.9	7.1	–	25. Participants (who)	8.2 (1.2)	86.2	13.8	–	24. Participants (who)	Ess.
25. Participant preparation	7.0 (1.6)	66.7	31.0	2.3	26. Participant preparation	7.1 (1.4)	60.7	39.3	–	25. Participant preparation	Suppl.
26. Methods used to deliver activities (how)	8.0 (1.2)	85.7	14.3	–	27. Methods used to deliver activities (how)	8 (1.2)	82.8	17.2	–	26. Methods used to deliver activities (how)	Ess.
27. Efforts to ensure fidelity of participants	7.7 (1.3)	82.9	17.1	–	28. Efforts to increase and sustain participation	7.6 (1.3)	79.3	20.7	–	27. Efforts to increase and sustain participation	Ess.^c^
28. Efforts to ensure fidelity of providers/staff	7.9 (1.1)	88.1	11.9	–	29. Efforts to ensure provider adherence to protocol	7.7 (1.2)	85.7	14.3	–	28. Efforts to ensure provider adherence to protocol	Ess.
					30. Monitoring of the programme implementation	7.6 (1.2)	82.1	17.9	–	29. Monitoring of the programme implementation	Ess.
29. Acceptability	7.9 (1.1)	88.1	11.9	–	31. Acceptability	7.3 (1.4)	72.4	27.6	–	30. Acceptability	Suppl.
30. Appropriateness	7.7 (1.2)	85.7	14.3	–	32. Appropriateness	7.2 (1.2)	69.0	31.0	–	31. Appropriateness	Suppl
31. Feasibility/Practicality	8.0 (1.1)	90.5	9.5	–	33. Feasibility/Practicality	7.9 (1.0)	89.7	10.3	–	32. Feasibility/Practicality	Ess.
32. Adoption	8.5 (1.0)	92.9	7.1	–	34. Adoption	8.2 (0.9)	96.6	3.5	–	33. Adoption	Ess.
33. Coverage/Reach	8.1 (1.1)	90.5	9.5	–	35. Coverage/Reach	8 (1.1)	93.1	6.9	–	34. Coverage/Reach	Ess.
34. Attrition	8.1 (1.1)	88.1	11.9	–	36. Attrition	7.7 (1.1)	82.8	17.2	–	35. Attrition	Ess.
35. Unexpected end of programme	8.5 (0.8)	97.6	2.4	–	37. Unexpected end of programme	8.1 (1.2)	89.3	10.7	–	36. Unexpected end of programme	Ess,
36. Reversibility	7.1 (1.3)	65.8	34.2	–	38. Reversibility	6.5 (1.3)	46.2	53.9	–	37. Reversibility	Suppl.
37. Contamination of activities	7.3 (1.4)	69.1	30.9	–	39. Contamination of activities	7.3 (1.0)	74.1	25.9	–	*Merged with #35*	
38. Fidelity	8.2 (1.0)	90.2	9.8	–	40. Fidelity	8.4 (0.9)	92.9	7.1	–	38. Fidelity	Ess.
39. Reasons for low fidelity	7.9 (1.1)	88.1	11.9	–	41. Reasons for low fidelity	7.9 (1.1)	85.2	14.8	–	*Merged with #38*	
40. Sustainability	8.3 (1.1)	88.1	11.9	–	*Moved to #49*						
41. Costs of implementation	8.2 (1.0)	92.9	7.1	–	42. Costs of implementation	8.1 (1.1)	93.1	6.9	–	39. Implementation costs/resources	Ess.
*Programme Evaluation*				*–*	*Programme Evaluation*				*–*	*Programme Evaluation*	
42. Process evaluation methods	8.5 (0.9)	95.1	4.9	–	43. Process evaluation methods	8.3 (1.0)	89.7	10.3	–	40. Process evaluation methods	Ess.
43. Effect of implementation process on results	8.3 (1.0)	95.0	5.0	–	44. Effect of implementation process on results	8.1 (1.0)	89.7	10.3	–	41. Effect of implementation process on results	Ess.
44. External events affecting implementation	8.1 (0.9)	95.1	4.9	–	45. Factors affecting implementation	8.2 (0.9)	93.1	6.9	–	42. Factors affecting implementation	Ess.
45. Ethical considerations	8.2 (0.9)	95.1	4.9	–	*Moved to #11*						
46. Implementation barriers and facilitators	8.1 (0.9)	97.6	2.4	–	*Moved to #50*						
47. Strengths and limitations	8.2 (0.9)	95.2	4.8	–	*Moved to #50*						
48. Outcome evaluation methods	8.5 (1.2)	90.5	9.5	–	46. Outcome evaluation methods	8.4 (0.8)	96.6	3.5	–	43. Outcome evaluation methods	Ess.
49. Unexpected/negative effects	8.3 (0.8)	97.6	2.4	–	47. Unexpected programme effects	8.2 (0.9)	93.1	6.9	–	44. Unexpected programme effects	Ess.
50. Differential effects	8.0 (1.1)	88.1	11.9	–	48. Differential effects	8.2 (1)	89.7	10.3	–	45. Differential effects	Ess.
					49. Sustainability	7.8 (0.9)	92.6	7.4	–	46. Sustainability	Ess.
					50. Strengths and limitations (lessons learnt)	8.4 (0.9)	96.4	3.6	–	47. Strengths and limitations (lessons learnt)	Ess.

^a^Essential (*Ess*.), Important (*Imp*.), Not important (*Not imp*)

^b^For round 3, essential items had to be marked as essential for the PRS by at least 80% of participants, while items below this cut-point could be considered as supplementary items

^c^Items ranked as essential by 70–79% of participants were considered b*orderline* essential

The ranking of most items remained high in round 2 and none were rejected. Based on participants’ comments we merged six items generating a revised list of 47 items, out of which 28 received high consensus as essentially important for a PRS tool. Two items were ranked as borderline essential (79% agreement).

In the third and final round, all but one (27 of 28) of the items ranked as essential (scores 7–9) in round 2 received high consensus (80%) that this was the correct categorization, and three items were borderline essential. No items were rejected and we did not make additional changes to the items prior to the technical consultation. A detailed overview of the Delphi item scores and suggestions is available in Additional file 2.

#### Overarching issues raised by the Delphi participants

Beyond the suggestions for additional items, we identified several general issues raised by the participants in the open-ended comment sections. These comments were grouped into four main categories across the three survey rounds: 1) *Clarification of items*, 2) *Justification of rankings*, 3) *Purpose of PRS development* and 4) *Programme results.*

Comments related to the *clarification of items* focused on the need to elaborate or better describe the meaning of specific items, or that its applicability might depend on the programme, as in these examples:

*Theory/logic model. These things aren't meaningful to everyone. More background/rationale rather than an important component of implementation.*

*The definition of Sustainability is a little odd. Would it be clearer to say something along the lines of 'the ability to maintain the programme and its effects over time?*

Some items were revised accordingly, while others will be further elaborated in a WHO document that will describe and explain how to use the PRS.

A number of participants also made comments to *justify their rankings*, noting the difficulty in ranking items as they “all are important and useful”.

*The relevance of Fidelity depends on the circumstances. If one is conducting a study, you need to be able to determine if you have poorer than expected performance whether the problem is one of design or execution. So, in that setting, knowledge of fidelity is important.*

Some participants posed questions about the *purpose of the PRS* and how it will be used; for example, whether items should be used to describe pilots or on-going programmes.

*I've found it somewhat difficult responding to questions, not having a clear enough sense of what's meant by "program" and, when "reporting" is referred to, who is to be reporting to whom? In places, the language suggests that what's referred to is some kind of pilot effort. In some places, the language suggests some short-term "intervention" (like a training activity). In other places, it sounds more like on-going service delivery.*

There was also feedback related to items missing to describe *programme results*, for example by asking where “anticipated/expected effects are reported” given that the evaluation domain “doesn't seem to include reporting of the impacts apart from unexpected/negative effects and differential effects”. While the original purpose of the PRS tool was to provide guidance for the reporting of programme development, implementation and evaluation *processes* rather than results, this issue was further discussed during the technical consultation as described below.

### Technical consultation results

The technical consultation to further refine the PRS brought together experts representing 17 non-governmental, governmental, bilateral and multilateral organizations from 12 countries in different global regions (Table 3). The synthesized feedback from this interactive expert meeting resulted in a number of changes to the content and description of items. Most importantly, the groups’ feedback resulted in substantial improvements to the structure and user-friendliness of the PRS by re-organizing and merging items (sometimes with new headings), and rewording many of research-oriented items to make these more programme-oriented. A decision was also made to use sub-items (1a, 1b, 2a, 2b…) to further improve the flow and structure of the PRS rather than numbering each item. Almost all of the items that received high consensus as essential during the Delphi survey were kept in some form, but their structure and wording changed or they were merged with other items, and most supplementary items were integrated into the revised list. For example, the items *Target population and area* (essential) and *Partners and stakeholder involvement* (supplementary) were included as sub-items under a new item called *Stakeholders*. The item *Components/activities* (essential) was expanded to cover the items *Timing* (borderline essential)*, Setting* (essential)*, Dose and intensity* (essential), *Provider/staff characteristics* (supplementary)*, Provider/staff training* (supplementary)*, Participants* (essential)*,* and *Methods used to deliver activities* (essential). Some supplementary items (e.g. *Programme name, Dissemination plans, Appropriateness, Participant preparation*) were deleted as the group perceived these to be redundant. As a result of the revised structure, all items were considered by the expert group to be essential, thus removing the need to distinguish between essential and supplementary items.

**Table 3:** Organizations represented in the PRS Technical Consultation (in alphabetical order)

Organization	Country*
African Population and Health Research Center (APHRC)	Kenya
Aga Khan Development Network	France
BBC Media Action	United Kingdom
Gynuity Health Projects	USA
ICDDR, B	Bangladesh
Institute of Population and Public Health	Canada
Institute of Tropical Medicine	Belgium
International Federation of Red Cross and Red Crescent Societies	Switzerland
MaiMwana Project	Malawi
Maternal and Child Survival Program	USA
Packard Foundation	Pakistan
Pathfinder International	USA
Population Council	Kenya
Population Sciences International (PSI)	Zambia
Rutgers	Netherlands
U.S. Agency for International Development (USAID)	USA
United Nations Population Fund (UNFPA)	Multilateral
WHO, Alliance for Health Policy and Systems Research	Multilateral
WHO, Department of Maternal, Newborn, Child and Adolescent Health	Multilateral
WHO, Department of Reproductive Health and Research	Multilateral

*For organizations operating globally such as Packard Foundation, Pathfinder International, Population Council, PSI etc, the country origin of the technical consultation participant has been reported

Much attention was given to the role of context in programme reporting, and as a result, the expert group decided that context should be added as a specific item as well as highlighted where applicable throughout the PRS. The group also discussed the need for the PRS to capture the dynamic nature of many programmes, including how and why activities change over time. Following up on the issue of results raised in the Delphi survey, the group further decided that *Results* should indeed be part of the PRS and reported together with the programme evaluation process.

Taken together, the group condensed the Delphi version of the PRS, including 27 essential and 20 supplementary items, into 24 items across five re-organized domains: *Programme overview; Programme components and implementation; Monitoring of Implementation*; *Evaluation and Results*; and *Synthesis*. This version was piloted as further described below. A detailed overview of the versions prior to and following the technical consultation is available in Additional file 2.

### Piloting results

Key characteristics of the four programmes selected to participate in the pilot are summarized in Table 4. These included a media and communication programme (BBC Media Action); a maternal health programme (United Nations Population Fund [UNFPA]); a family planning service delivery programme (United States Agency for International Development [USAID]); and a SRH information and service delivery programme (Rutgers).

**Table 4:** Overview of programmes that participated in the piloting of the draft PRS

Organization	Country	SRMNCAH topic covered	Type of programme	Scale	Duration
BBC Media Action	Somalia	Maternal, neonatal and child health	Social and behaviour change communication	National	3 years
UNFPA	Afghanistan	Reproductive, maternal and adolescent health	Prevention, treatment and social integration services	Global (one country selected)	5 years
USAID	Jordan	Sexual and reproductive health	Service delivery (private and non-governmental partnerships to expand family planning access)	National	5 years
Rutgers	Kenya	Sexual and reproductive health	Service and information delivery and uptake related to sexual and reproductive health	Global (one country selected)	5 years

Overall, the reviewers were able to report on and provide the source of all items applicable to the programme. While the second reviewer verified most of the items, there was some disagreement on whether or not certain items were reported on, mainly due to differential interpretations of their meaning and how much information should be provided in order for an item to be considered “reported”. Indeed, reviewers raised the questions of how extensive the items should be reported, highlighting the tension between *what* is reported and the *quality* of such reporting.

*I also found it hard not to want to give a qualitative assessment of the information provided on each item, i.e. where there was some information reported on an item but I didn’t feel the reporting was complete/of a high standard. Guidance on how to approach partial/weak reporting on certain items when completing the tool would be helpful.*

In their comments, all reviewers highlighted the usefulness of the tool to both guide programme reporting as well as to strengthen up-front programme design. However, they also stressed that the PRS took longer to complete than they had anticipated. This was mainly due to the fact that the information provided was scattered between multiple different sources and rarely were all items reflected in one single report. Despite its length, none of the reviewers felt the need to remove items, but rather noted that the PRS was useful to help them organize different information sources, and that it might be easier to use in a prospective manner.

The reviewers further emphasized that the PRS may need to be applied differently to large-scale programmes consisting of multiple different components. As one reviewer noted:

*If this list will become the norm of reporting, it means the reports will remain many and big for such a large programme, whereas this is not always considered useful. Currently, there is even a tendency from our donor to require smaller reports, mostly based on reflection. Therefore, it should be clear how reports based on this PRS tool will be used.*

This issue was further discussed during the virtual meeting during which the reviewers agreed that larger programmes might need to be broken down into smaller components (e.g. country levels, or specific topics) as part of the reporting process.

None of the items were deleted as a result of the piloting; minor language edits were made to further clarify the description and flow of the 24 items; and decisions were made as to what information should be picked up in the WHO document to be developed where more in-depth explanations will be made available.

### PRS version 1.0

Table 5 presents the PRS (version 1.0), which consists of 24 items across five domains: 1) programme overview, 2) programme components and implementation, 3) monitoring of implementation, 4) evaluation and results, and 5) synthesis. Below we provide a brief description of each domain.

**Table 5:** Programme reporting standards (PRS) for SRMNCAH – version 1.0

The PRS is a tool that can be used for reporting on the planning, implementation and evaluation of SRMNCAH programmes. The PRS can be used throughout the programme lifecycle, guiding not only the reporting of processes and outcomes but also the programme design and development. Instructions for using the PRS • For each reporting item, provide the source and page number where the information can be located. • If the information provided for an item is deemed insufficient, state “not reported”. • While users of the PRS should consider the relevance of all items, some items may not be applicable to the programme or the specific report. If an item is irrelevant or beyond the scope of the programme, indicate “N/A” • Larger programmes may need to break their reporting into more specific components and topics.		
Section and item name	Item description	Reported (source and page) Not reported N/A
Programme Overview	*Why was the programme started and what did it expect to achieve?*	
1. Rationale and objectives	a. Programme rationale, i.e. why the programme was initiated (nature and significance of the issue or problem being addressed).	
	b. Goals and objectives.	
	c. Anticipated short- and long-term effects of the programme at different levels (e.g. individual, household, facility, organization, community, society).	
2. Start and end date	a. Planned start and end date of the programme.	
	b. Delays and/or unexpected end of the programme along with reasons why.	
3. Setting and Context	a. Where the programme took place, e.g. country name(s), specific locations, urban/rural environments.	
	b. Overview of the context (e.g. political, historical, sociocultural, socioeconomic, ethical, legal, health system) pertinent to the programme.	
4. Stakeholders	a. Programme target population (key sociodemographic characteristics e.g. age, gender, ethnicity, education level)	
	b. Implementing organization(s).	
	c. Partners and other stakeholders (e.g. local authorities, community leaders).	
	d. How the different stakeholders were involved in programme development and/or implementation.	
5. Funding source(s)	Name of programme donor/funding source(s).	
6. Theory of change and/or logic model	Theory of change, assumptions, and/or logic model framework underlying the programme, with details for how this guided the programme design, implementation and evaluation plans.	
7. Human rights perspectives	a. If and how gender, equity, rights and ethical considerations were integrated into the programme.	
	b. If and how an accountability framework was adapted to define the programme’s commitments and how it would be accountable for these commitments.	
Programme Components and Implementation	*What did the programme do and how?*	
8. Programme planning	How activities were decided upon and why (e.g. based on results of a situational or stakeholder analysis, identification of current gaps and needs in programming, or criteria such as the evidence-base, scalability, sustainability of activities).	
9. Piloting	Piloting of the programme activities elsewhere or within the programme, and if so how, when, where, by whom and with what results.	
10. Components/Activities (Please repeat for each component)	Detailed description of the core programme components/activities, including: • *What* was done • *How* (implementation methods/delivery processes/approaches). • *When* (frequency, intensity, duration). • *By whom* (characteristics, skills, training and responsibilities of implementing personnel (i.e. staff, providers, volunteers). • *For whom* (target population for each activity). • Support materials used and where these can be accessed.	
11. Quality assurance mechanisms	a. Mechanisms used to ensure the quality in the implementation of activities (e.g. supervision and support of personnel, refresher trainings, product quality checks).	
	b. Efforts used to increase and sustain participation of stakeholders (e.g. incentives).	
Monitoring of Implementation	*How did the programme keep track of what was done?*	
12. Monitoring mechanisms	How the programme implementation process was monitored, including the collection and analysis of indicators to identify problems/solutions.	
13. Coverage/Reach and Drop-out	a. Uptake (utilization) each programme activity reported by key sociodemographics characteristics.	
	b. Coverage of the programme activities, including differential reach in or outside of the target population.	
	c. Non-participation and dropout among the target populations, along with key sociodemographics and reasons for why.	
14. Adaptations	a. Whether the programme was delivered as intended, e.g. discrepancies between programme design vs. the actual implementation of components, degree of match between programme content and theory of change.	
	b. On-going adaptation of the programme activities to better fit the context, and the fidelity to the activity plan.	
15. Acceptability	Acceptability of the programme among stakeholders, e.g. assessment of whether the programme was considered to be reasonable and relevant.	
16. Feasibility	Assessment of the feasibility of the programme, e.g., the extent to which it could be carried out in the particular context or by the specific organization.	
17. Factors affecting implementation	Description of key barriers and facilitators to programme implementation, including contextual factors (e.g. social, political, economic, health systems).	
Evaluation and Results	*How was the programme evaluated, and what were the findings?*	
18. Evaluation	a. Type of evaluation(s) conducted (e.g. process evaluation and/or outcome evaluation, quantitative or qualitative).	
	b. Evaluation methods. How, when (timing and phases e.g. baseline, midline, end line) and by who the programme was evaluated^a^.	
19. Results	a. Description of the programme results (key process, output, outcome indicators), differentiating between short/mid/long-term effects.	
	b. Whether the programme effects differed across key sociodemographic characteristics and/or geographical areas.	
	c. Whether the programme had unexpected effects (beyond what was anticipated in the design) on the target population, health services and/or the communities	
20. Costs	a. Summary of the required resources for implementation (e.g., financial, time, human resources, materials, administration)	
	b. If and how a cost analysis or cost-effectiveness analysis was conducted.	
Synthesis	*What are the key implications?*	
21. Lessons learnt	Appraised weaknesses and strengths of the programme, what worked well and what can be improved.	
22. Sustainability	Reflections on the sustainability of the programme over time, e.g. the expected ability to maintain the programme activities, engagement of stakeholders, outcomes achieved, effects, partnerships.	
23. Scalability	Description of the scale-up of all or some programmes activities, or any plans for scale-up.	
24. Possibilities for implementation in other settings	Reflections on the context-dependence of the programme and (and with what degree of effort) it could be implemented in/adapted to other settings.	
Additional information (optional)	References and/or links to additional sources of information in relation to the programme.	
	Any additional comments related to the items reported above	

^a^Reports of research studies should provide further details in line with guidelines for the reporting of the specific study design. Different guidelines are available in the EQUATOR database (http://www.equator-network.org/)

#### Programme overview

The programme overview section consists of seven items, starting with the programme’s rationale and objectives (why it was initiated, goals, anticipated effects), start and end date, and the programme context (geographical setting and an overview sociocultural, political, historical, legal, health systems or other relevant contextual aspects). Additionally, this domain includes a description of the programme stakeholders and their roles, the funding source, use of a theory of change or logic model to guide the programme, and human rights perspectives (e.g. gender, equity, rights and ethical considerations) as well as the use of an accountability framework to define and follow up on such commitments.

#### Programme components and implementation

Four items make up the programme components and implementation section, starting with how activities were decided upon and why, any piloting of activities along with results, followed by a detailed description of each core component, including what was done, how, when, by whom, for whom, and any support materials used. This section also includes an overview of mechanisms for ensuring the quality of implementation such as support to personnel, refresher trainings and product quality checks, and any efforts to increase and sustain the participation of different stakeholders.

#### Monitoring of Implementation

The third section covers six items related to how the programme implementation was monitored (and indicators used to track progress), the coverage and reach of activities, any adaptations to the programme (whether it was delivered as intended, on-going adaptation of the activities to better fit the context), and reflections on whether the programme was considered to be reasonable and relevant among stakeholders. The section ends with a reflection on the feasibility of the programme (i.e. the extent to which the it could be carried out the context where it was implemented), and *factors affecting implementation* (key barriers and facilitators)..

#### Evaluation and results

The fourth section covers three items related to whether an *evaluation* was conducted (type of evaluation and specific methods), and a description of the programme *results* including key indicators (process, output, outcome), short, mid and long-term effects by key sociodemographic characteristics as well as potential unexpected effects, and an overview of the required resources for implementation and whether a cost analysis or cost-effectiveness analysis was conducted.

#### Synthesis

The final section of the PRS includes four items to describe the key lessons learnt, the sustainability (e.g. expected ability to maintain activities) as well as scalability of the programme, and a reflection on the context-dependence of the programme (whether it could be implemented in or adapted to other settings).

At the end of the PRS, a section is included to allow for supplemental information (e.g. references/links to relevant sources), and any additional comments that could help clarify the items reported.

## Discussion

In this paper we present the PRS 1.0; a tool intended to guide, and thus aims to improve the reporting of SRMNCAH programme design, implementation, monitoring and evaluation processes. The PRS is the result of a structured, collaborative work process including interactive input from different stakeholders in the field of SRMNCAH. The 24 items included in the PRS reflect those identified to be most central to the adequate and transparent reporting of programmes. The overarching goal of the PRS is to facilitate exchange of information and evidence synthesis within and between different programmes and sectors working to improve the health and wellbeing of individuals across the SRMNCAH continuum. Our efforts are in line with the increased recognition to understand not only the evidence-base of outcomes but how and in what contexts successful outcomes were achieved [3, 4]; what worked and what did not work, challenges faced in the field and actions taken to overcome such challenges.

In particular, the PRS responds to the need for more accurate descriptions of the role of context in programme development and implementation [3–5]. While its interpretation and meaning vary greatly in health sciences, a recent review defined context as “a set of characteristics and circumstances that consist of active and unique factors that surround the implementation effort” [5]. Central to this definition is the fact that context is not just a “backdrop” of programme implementation but “interacts, influences, modifies and facilitates or constrains the intervention and the implementation effort” [5]. Indeed, the complex and multifaceted nature of context and how best to capture this as part of programme reporting was intensely debated throughout the PRS development. In particular, participants in the Delphi survey as well as technical consultation noted the tension between including a specific item to describe programme context versus integrating the role of context throughout the PRS. This deliberation resulted in the decision to do both; i.e. the PRS includes both a specific item that provides an overview of the programme context (item 3), but the role of contextual elements in shaping programme implementation is also highlighted throughout the PRS, for example in items 16 (feasibility), 17 (factors affecting implementation) and 24 (possibilities for adaptation in other settings).

A central challenge has been to develop PRS so that is broad enough to apply to a wide range of fields within the SRMNCAH continuum; yet specific enough so that people feel it is useful for and applies to their specific programme or topic area. Similarly to the WHO Bulletin *Reporting guidelines for implementation and operational research* [2], the PRS is not specific to a certain programme or study design, but rather integrates and builds upon existing tools. Given the many checklists have already been developed for reporting of specific research designs in the peer-reviewed journals, we feel that this does not need to be repeated in the PRS. Rather, programme staff and authors of reports and publications should use guidelines applicable to their selected design for the programme evaluation while reporting them for publications. Beyond the designs listed in Additional file 1, the Enhancing the QUAlity and Transparency of Health Research (EQUATOR) network provides an excellent overview of research reporting guidelines (http://www.equator-network.org/library/) according to different designs and topics.

An additional question raised is *when* the PRS should be used. While the piloting conducted as part of this paper was retrospective and provided a sense of whether the items could be located in existing programme reports, our intent is that the PRS will be used in a prospective manner to guide the continuous reporting of programmes. In addition, many of the items might be highly relevant to consider at the beginning of the programme; i.e. “beginning with the end in mind” [11]. As part of the verification process, we noted how the reported items were scattered between multiple different reports and other sources (e.g. proposals, evaluations, briefs, monitoring frameworks) and the goal is thus to provide support for how this information can be brought together. In this respect the PRS is conceptually different from other research reporting standards.

The PRS can be used to note where additional information can be located beyond what is written in any report or publication. Furthermore, because of its comprehensive nature and amount of detail covered by the items, larger programmes may break their reporting into smaller components. For example, a global programme could be broken into national level or to specific sub-topics or objectives in a way that does not increase but rather ease the “burden” of reporting. It is also important to note that reported information does not need to be organized in the exact same order as the tool is structured; the intent is to use PRS is a checklist to verify that the core items have been covered.

What, then, “counts” as an item being covered or reported on? A key issue highlighted by our respondents was how to judge whether the information provided is sufficient and of good enough quality to be considered reported. Importantly, the PRS is not a quality assessment framework, but a tool to guide the reporting of programmes with specific focus on context and implementation processes. While there is no gold standard for how much information to provide, a sufficient amount should however be provided to allow for someone not familiar with the programme to understand the context and rationale underlying its implementation. In this first version of the PRS, we have agreed on what items need to be reported. How much information is needed, and the quality of that information, will be further assessed through the continuous use of the tool where feedback from the field will be essential. The PRS will be available to the public through the WHO website as well as the website of key partners. In addition, the WHO will develop a guidance document that explains and elaborates on each item in greater detail. The WHO also intends to actively use this tool as part of on-going initiatives and implementation, for example by sharing country experiences in a structured way as part of the learning platform designed for improving quality of maternal and newborn care in health facilities [15], as well as to promote its use across implementing and donor organizations. Many of the key partners and donors who have been part of the PRS development have also committed to actively promoting its use with the programmes that they support. Our goal is to follow up with agencies using the PRS during 2018 to determine what modifications could be made for version 2.0.

### Strengths and limitations

In line with recommendations for the development of reporting guidelines [11], we developed the PRS through a systematic approach that is grounded both in the synthesis of existing literature as well as expert opinions and a piloting process. A key strength was the use of a Delphi panel with experts from different organizations in the field of SRMNCAH to rank and reach consensus on core reporting items. While the Delphi process was useful for us to prioritize the 50 items identified in the systematic review and invite feedback from a larger group, the technical consultation with a smaller group of experts provided a more in-depth understanding and feedback, which was not feasible during the Delphi. This meeting was essential to the development of the PRS; in fact, it was during this process that most changes to the PRS were made as a result of the interactive discussions, informed by the Delphi rankings. The revisions following the technical consultation were further evaluated in the pilot, which allowed more specific input from staff and managers with regards to the feasibility and applicability of the PRS to their specific programmes.

The development of the PRS should also be considered in light of its limitations. First, the consultative process was limited to the people who responded to the Delphi and came to the technical consultation. Although the PRS may be applied more generically, we developed the tool with input from the SRMNCAH field. In addition, the draft PRS was only piloted retrospectively with four existing programmes and it is possible that we would have received more input related to the real-word utility and been able to address issues prior to the launch of the PRS had we been able to extend this initial piloting face to a larger number of interventions, and over time. As such, it will be important to continuously seek input and update the PRS to reflect input from a wider audience.

## Conclusion

The starting point for the PRS development was the need for better reporting of why, how, when and under which circumstances programme activities are implemented, including lessons learnt from the field. We believe that the PRS will be a useful reference to programme implementers and researchers in the field of SRMNCAH. As implicit by its name – PRS 1.0 – this is the first version of the tool. The development of the PRS is an evolving process, and we look forward to input from the field to improve its relevance and usefulness.

## Additional files

Additional file 1: Reporting guidelines for research studies. (DOCX 108 kb)

Additional file 2: Summary of results of the systematic review, three-step Delphi process and the draft PRS in preparation for the technical consultation. (DOCX 565 kb)

## Abbreviations

EQUATOR: Enhancing the QUAlity and Transparency of Health Research
HRP: UNDP/UNFPA/UNICEF/WHO/World Bank Special Programme of Research, Development and Research Training in Human Reproduction
PRS: Programme Reporting Standards
SRH: Sexual and Reproductive Health
SRMNCAH: Sexual, reproductive, maternal, newborn, child and adolescent health
StaRI: Standards for Reporting Implementation Studies of Complex Interventions
TIDieR: Template for Intervention Description and Replication
UNFPA: United Nations Population Fund
USAID: United States Agency for International Development
WHO: World Health Organization
