# Supplementary material for: Programme Reporting Standards (PRS) for improving the reporting of sexual, reproductive, maternal, newborn, child and adolescent health programmes
Source: BMC Med Res Methodol. 2017 Aug 3;17:117. doi: 10.1186/s12874-017-0384-7 (PMC5543449; doi:10.1186/s12874-017-0384-7)
Supplement: Supplementary file 1 — Reporting guidelines for research studies. (DOCX 108 kb) [file 12874_2017_384_MOESM1_ESM.docx]

**Additional File 1. Reporting guidelines for research studies***

| **Type of study** | **Guideline name** | **Reference** |
| --- | --- | --- |
| Randomized trials | Consolidated Standards of Reporting Trials (CONSORT) (with extensions) | [1] |
| Intervention description and replication | Template for Intervention Description and Replication (TIDieR) | [2] |
| Public health, health systems and policy interventions | UNTIDieR (under development) | [3] |
| Observational studies | STrengthening the Reporting of OBservational studies in Epidemiology (STROBE) (with extensions) | [4] |
| Non-randomized interventions | Template for intervention description and replication (TREND) | [5] |
| Operational research | Reporting guidelines for implementation and operational research | [6] |
| Implementation studies on complex interventions | Standards for Reporting Implementation studies of complex interventions (StaRI) | [7] |
| Case reports | CAse REport guidelines (CARE) (with extensions) |  |
| Qualitative research | Standards for Reporting on Qualitative Research (SRQR) (with extensions) | [8] |
| Quality improvement studies | Standards for Quality Improvement Reporting Excellence (SQUIRE) | [9] |
| Economic evaluations | Consolidated Health Economic Evaluation Reporting Standards (CHEERS) | [10] |
| Diagnostic/prognostic studies | Standards for reporting on diagnostic accuracy studies (STARD) | [11] |
| Systematic reviews | Preferred reporting standards in systematic reviews and meta-analyses (PRISMA) | [12] |

*Adapted from hhtp://equator-network.org and Hales et al [6].

**REFERENCES**

1. Altman DG, Schulz KF, Moher D, Egger M, Davidoff F, Elbourne D, et al. The revised CONSORT statement for reporting randomized trials: explanation and elaboration. Ann Intern Med. 2001;134: 663-694.

2. Hoffmann TC, Glasziou PP, Boutron I, Milne R, Perera R, Moher D, et al. Better reporting of interventions: Template for intervention description and replication (TIDieR) checklist and guide. BMJ (Online). 2014;348.

3. EQUATOR network. UNTIDieR project summary. Available: <http://www.equator-network.org/wp-content/uploads/2009/02/UNTIDieR-project-summary.pdfl>

4. Vandenbroucke JP, von Elm E, Altman DG, Gotzsche PC, Mulrow CD, Pocock SJ, et al. Strengthening the Reporting of Observational Studies in Epidemiology (STROBE): explanation and elaboration. PLoS Med. 2007;4: e297.

5. Des Jarlais DC, Lyles C, Crepaz N. Improving the reporting quality of nonrandomized evaluations of behavioral and public health interventions: the TREND statement. Am J Public Health. 2004;94: 361-366.

6. Hales S, Lesher-Trevino A, Ford N, Maher D, Ramsay A, Tran N. Reporting guidelines for implementation and operational research. Bull World Health Organ. 2016;94: 58-64.

7. Pinnock H, Epiphaniou E, Sheikh A, Griffiths C, Eldridge S, Craig P, et al. Developing standards for reporting implementation studies of complex interventions (StaRI): a systematic review and e-Delphi. Implementation Science. 2015;10: 42.

8. O'Brien BC, Harris IB, Beckman TJ, Reed DA, Cook DA. Standards for reporting qualitative research: a synthesis of recommendations. Acad Med. 2014;89: 1245-1251.

9. Ogrinc G, Mooney SE, Estrada C, Foster T, Goldmann D, Hall LW, et al. The SQUIRE (Standards for QUality Improvement Reporting Excellence) guidelines for quality improvement reporting: explanation and elaboration. Qual Saf Health Care. 2008;17 Suppl 1: i13-32.

10. Husereau D, Drummond M, Petrou S, Carswell C, Moher D, Greenberg D, et al. Consolidated Health Economic Evaluation Reporting Standards (CHEERS) statement. Value Health. 2013;16: e1-5.

11. Bossuyt PM, Reitsma JB, Bruns DE, Gatsonis CA, Glasziou PP, Irwig LM, et al. The STARD statement for reporting studies of diagnostic accuracy: explanation and elaboration. Ann Intern Med. 2003;138: W1-12.

12. Liberati A, Altman DG, Tetzlaff J, Mulrow C, Gotzsche PC, Ioannidis JP, et al. The PRISMA statement for reporting systematic reviews and meta-analyses of studies that evaluate health care interventions: explanation and elaboration. PLoS Med. 2009;6: e1000100.
