# Supplementary material for: Programme Reporting Standards (PRS) for improving the reporting of sexual, reproductive, maternal, newborn, child and adolescent health programmes
Source: BMC Med Res Methodol. 2017 Aug 3;17:117. doi: 10.1186/s12874-017-0384-7 (PMC5543449; doi:10.1186/s12874-017-0384-7)
Supplement: Supplementary file 2 — Summary of results of the systematic review, three-step Delphi process and the draft PRS in preparation for the technical consultation. (DOCX 565 kb) [file 12874_2017_384_MOESM2_ESM.docx]

**Additional file 2.** Document summarizing the results of the systematic review, three-step Delphi process and the draft PRS in preparation for the technical consultation

**Table of Contents**

**Introduction 2**

**Systematic review to identify core reporting items 3**

**Delphi exercise to reach consensus on core PRS items 3**

**Delphi participants and procedure 3**

Rounds 1 and 2 4

Round 3 4

Analysis 5

**Delphi survey results 5**

Participant characteristics 5

Rounds 1 and 2: item rankings and suggestions 5

Consensus following round 3 6

Participant comments 6

**Other feedback on draft PRS tool 8**

**Technical consultation to refine and finalize draft PRS 9**

**Tables 10**

**References 29**

**Introduction**

Adequate and transparent reporting about programme processes is key to understanding their impact as well as guide successful replication and scale up. However, the reality is that many sexual, reproductive, maternal, newborn, child and adolescent health (SR/MNCAH) programmes operate under complex, real-world conditions which often make it difficult to communicate exactly what is being done, when, where, how and by whom in a timely and consistent manner. In order for others to learn from their experiences, programme coordinators, implementers, managers as well as researchers thus need a standardized way of reporting on processes throughout the programme.

Furthermore, the impact of many interventions particularly the social and behavioural/community engagement interventions is very much tied to the context and the processes of implementation. In order to assess the evidence on the effectiveness of interventions, expert groups require a better understanding of programme implementation and context, a standardized way of reporting on context and processes throughout the programme, would allow for easier synthesis of this information.

In response to this gap, WHO/RHR initiated a consultative process to develop Programme Reporting Standards (PRS) to be used by programme implementers and researchers in the field of SR/MNCAH. The project is a partnership between the WHO Department of Reproductive Health and Research, including UNDP/UNFPA/UNICEF/WHO/World Bank Special Programme of Research, Development and Research Training in Human Reproduction (HRP) and the Alliance for Health Policy and Systems Research. The overarching goal of the PRS is to provide guidance for complete and accurate reporting on the development, implementation and monitoring and evaluation processes of SR/MNCAH programmes.

The PRS tool is being developed in line with recommendations for developing reporting guidelines [1], including:

1. **Systematic review of** existing reporting tools applicable to sexual and reproductive health programmes.
2. **Delphi consensus survey** to revise and further refine the list of items by identifying the items of highest relevance for a PRS tool.
3. **Technical consultation** with experts to finalize the PRS based on the results from the systematic review and Delphi survey.
4. Following this meeting, the revised version of the PRS will be available for **piloting** **and user-testing by programmes** in the field of SR/MNCAH.

This background paper briefly presents the findings from the systematic review, followed by a more detailed overview of the results from the Delphi survey.

**Systematic review to identify core reporting items**

We conducted a systematic review of reporting guidelines, checklists and other tools, applicable for reporting on programmes targeting sexual and reproductive health outcomes (time period 2000-2014). Two independent reviewers screened the title and abstracts, and assessed full texts in duplicate, followed by data extraction (focus, content area, and description of reporting items). Thematic analysis was used to synthesize reporting items across the included tools, by extracting and aggregating items related to programme preparation, implementation and evaluation into a consolidated list.

Following the screening of over 3600 records, 74 articles corresponding to 45 reporting tools were included. A total of 50 reporting items across three main domains and subdomains were identified: programme preparation (objective/focus, design, piloting); programme implementation (content, timing/duration/location, providers/staff, participants, delivery, implementation outcomes), and programme evaluation (process evaluation, implementation barriers/facilitators, outcome/impact evaluation). The items are shown in Table 1. A detailed description of the review, including the methods (screening, data extraction and synthesis) are described in the publication that is attached to this background paper [2].

While we initially set out to develop a reporting tool specific to sexual and reproductive health programmes, the systematic review captured reporting tools related to diverse health programmes. All of the identified items were applicable to programmes within the broader frame of SR/MNCAH, and we therefore decided to expand the subsequent steps of the tool development to this area.

**Delphi exercise to reach consensus on core PRS items**

A three-round Delphi survey was conducted with experts in the field of SR/MNCAH to refine and revise the list of items generated in the systematic review by identifying those of highest relevance for a PRS tool. The Delphi survey technique (Delphi for short) is a method to explore, seek consensus and correlate judgments on a specific topic [3]. The method seeks the opinion of experts through an iterative series of structured survey rounds. Throughout the process, the responses and feedback from participants is fed into the next round until consensus has been reached

**Delphi participants and procedure**

Eighty-one experts in the areas of implementation, research and funding of SRH programmes and/or reporting guideline development were invited to participate in the Delphi survey. The process consisted of three prospectively planned rounds, with the first round starting in September 2015 and the last round concluding in March 2016. Participants had about two to four weeks to respond to each survey round, and reminders were sent after two and three weeks, respectively. Participants were initially encouraged to complete all survey rounds, however invitations for the second and third rounds were restricted to those that responded to the previous round to ensure continuity in response. For each round, participants received personalized emails containing a link to an on-line survey administered through the electronic instrument *SurveyMonkey.*

**Rounds 1 and 2**

The first Delphi round was conducted between 21 September and 12 October 2015 to introduce participants to the list of items generated from the systematic review, generate initial rankings of the relevance of each item for a PRS tool, and to obtain suggestions for item revisions. Participants were asked to rate the relevance of the 50 items identified in the systematic review for potential inclusion in a PRS tool using a Likert-type scale ranging from 1 (not important) to 9 (essentially important). Because the initial list of items was not exhaustive and merely reflected items available in existing reporting tools, participants were encouraged to suggest new items as well as modification to the structure and language of items and their descriptions. Several free-text sections were available for participants to provide suggestions, feedback and comments on the items and their wordings.

Participants who completed the first round (N=48) were sent a summary of the results including the ranking of each items and a revised list of the items based on the open-ended feedback from group. In the second round, conducted between December 8 2015 and January 11 2016, these participants were invited to rank the importance of the revised list of items using the same 9-point scale. Participants were also invited to comment on the structure or language of items and their descriptions.

**Round 3**

Following the second round, scoring results were once more fed back to participants who remained in the study (N=31) together with an updated list of items based on the group feedback. The goal of the final round, conducted between March 2 and 22 2016, was to reach consensus on items to be included in the PRS tool as essential or supplementary. Based on the ranking from Round 2, all items were assigned into one of two categories. Participants were asked to indicate their agreement with this categorization of each item as:

- **Essential** (include in a PRS tool). These items were ranked as essential by at least 80% of participants in Round 2.
- **Supplementary** (could be a supplementary item in a PRS tool.) These items received lower consensus, but were still ranked as important in Round 2.

**Analysis**

For the first two rounds, descriptive statistics including mean (SD), median (IQR), minimum and maximum scores were calculated for each item, with missing values coded as zero. The scores of each item were allocated into three categories of importance: *not important* (scores 1-3); *important or desirable but not essential* (scores 4-6); and *essentially important* (scores 7-9). These categories were used to estimate the % agreement between participants, defined as the proportion of participants rating an item in the same category of importance.

Open-ended comments from participants were synthesized using thematic analysis. Suggestions for new items were categorized into the PRS domains and sub-domains identified in the systematic review; new sub-domains were created when applicable. Comments on the structure or wording of items were also reviewed and clustered into similar categories or themes.

Responses from round 3 were used to estimate the percent agreement between participants as the proportion of participants rating an item in the same category of importance (e.g. the % consistently ranking an item as essentially important in Rounds 2 and 3). An a-priori level of consensus was set to 80% agreement of items as essentially important. We encouraged participants to comment on their disagreement with the categorization of items (if applicable), and these comments were reviewed and clustered into similar categories or themes.

**Delphi survey results**

**Participant characteristics**

Of the 81 experts initially invited, 59% (N=48) responded to the first round of the Delphi exercise. Out of these, 67% (N=32) completed the second survey round and 44% (N=21) responded to all three rounds. **Table 2** shows the characteristics of the survey participants across the three rounds. Among those that completed all three rounds, 30% represented Universities and 30% NGOs, and the remaining worked for governmental donors (15%), UN organs (10%) and global donors (5%). About half of participants reported a background in research/academia (58%) and/or programme planning or implementation (47%), followed by management (42%) and medical/clinical (37%) areas. Most participants (60% in the final round) came from organizations that conduct global work, followed by Africa (20%), Southeast Asia and North America (5% respectively).

**Rounds 1 and 2: item rankings and suggestions**

**Table 3** presents an overview of the scoring of items in rounds 1 and 2, and the changes made to items following the first round.

***Round 1:*** Most items in the first round received high ratings with mean scores ranging from 8.79 (*Overall goal/objectives of programme*) to 6.19 (*Innovation*). No items were ranked as “not important” (received a mean score below 4). Out of the 50 items generated in the systematic review, 23 items received high consensus (ranked as essentially important by 80% or more of the participants). This was true for 4 out of 11 programme preparation items, 21 out of 30 implementation items, and all evaluation items (Table 2).

Several participants made suggestions for additional items in relation to each domain, and these suggestions were collapsed into themes:

- Programme preparation: *Background and rationale for targeted SRH issues, Landscape/situation analysis, Stakeholder involvement in programme design, Up-front definition of indicators, Ethical considerations of the design, Planned geographical level of scale*, and *Programme timeline.*
- Programme implementation: *Unanticipated implementation effects, Monitoring of the programme implementation,* and *Programme indicators.*
- Programme evaluation: *Community participation in the evaluation process, Programme dissemination, and Validity and reliability of results*.

Most open-ended suggestions were integrated into existing items in order to make these more specific, and some items were moved across domains. Several revisions were also made to the wording and description of items in line with participant comments. Two suggestions for new items stood out (*Monitoring of programme activities*, and *Dissemination plans*) and were therefore included as new items.

***Round 2:*** The ranking of most items remained high in round 2, with mean scores ranging from 8.7 (*Objectives and anticipated effects*) to 5.8 (*Program Manual*). Following participants’ comments, some items were merged for a revised list of 47 items, out of which 28 received high consensus as essentially important for a PRS tool. Two items were borderline essential (79% agreement). While all remaining items received mean scores above 5 (i.e. none were ranked as unimportant), their lower consensus indicates that they could be supplemental rather than core items.

**Consensus following round 3**

**Table 4** presents an overview of the item consensus following round 3, together with a description of each item. Taken together, all but one (27 of 28) of the items ranked as essential (scores 7-9) in Round 2 received high consensus (80%) that this was the correct categorization (Table 3) and three items were borderline essential. No items were rejected by the respondents, and no additional changes were made prior to the technical consultation.

**Participant comments**

Beyond the suggestions for additional items, a number of general issues were identified based on participants’ comments. These comments were grouped into four main categories across the three survey rounds: 1) *Clarification of items*, 2) *Justification of rankings*, 3) *Purpose of PRS development* and 4) *Programme results.*

***Clarification of items:*** Comments related to the clarification of items usually focused on the need to elaborate or better describe the meaning of specific items. For example, that terminologies such as *Theory/logic model* might not be meaningful to everyone and that its applicability “depends on the program”. Some items were revised accordingly, while others will be elaborated in a guidance document, which will describe and explain the items in the final PRS tool. Examples included:

*‘Theory/logic model. These things aren't meaningful to everyone. More background/rationale rather than an important component of implementation.’*

*‘The definition of Sustainability is a little odd. Would it be clearer to say something along the lines of 'the ability to maintain the programme and its effects over time?’*

***Justification of items:*** Some participants also made comments to *justify their rankings*, noting the difficulty in ranking items as these all seem important.

*‘As for logic model, I think it depends on the program, I agree its very important, but some programs get too bogged down trying to detail the model instead of simply explaining what they did and why. I don't think not have a logic model is a reason not to review a paper or program for value. It certainly helps if there is one - but it can be essential.’*

*‘The relevance of Fidelity depends on the circumstances. If one is conducting a study, you need to be able to determine if you have poorer than expected performance whether the problem is one of design or execution. So, in that setting, knowledge of fidelity is important.’*

***Purpose of PRS development:*** Comments related to the purpose of *PRS* posed questions about how this tool will be used; for example, whether items should be used to describe pilots or on-going programmes. It is important to note that the purpose of the Delphi exercise was to identify the most central items that apply both to short term projects and ongoing programmes. However, some items may not apply depending on the nature of the project or programme, and if so these do not need to be reported on.

*‘I've found it somewhat difficult responding to questions, not having a clear enough sense of what's meant by "program" and, when "reporting" is referred to, who is to be reporting to whom? In places, the language suggests that what's referred to is some kind of pilot effort. In some places, the language suggests some short-term "intervention" (like a training activity). In other places, it sounds more like ongoing service delivery.’*

*‘Many seem relevant for pilots or special studies, not on-going programs. I think the requirements for documentation and reporting differ. Not having a good sense of the referent in this case, I found it difficult to answer some of these questions.’*

***Programme results:*** Overall, many participants commented on the lack of an item to describe programme results. The original purpose of the PRS tool was to provide guidance for the reporting of programme development, implementation and evaluation *processes*, and the intent has so far not been to provide guidance on describing programme results. Given the many checklists that have already been developed for results reporting, the plan has been link the PRS tool with such existing guidelines. However, this issue requires further discussion during the technical consultation.

*‘Agree that Unexpected programme effects is essential, but wondering where anticipated/expected effects are reported?’*

*‘Impact/outcome evaluation: doesn't seem to include reporting of the impacts apart from unexpected/negative effects and differential effects?’*

**Other feedback on draft PRS tool**

In addition to the Delphi survey and technical consultation, the PRS development has been presented and discussed at international conferences, trainings and working-group meetings. Extensive feedback was received during a special side-session at the Social and Behavioral Change Communication (SBCC) Summit held in Addis Ababa during February 2016. Following a presentation on the PRS, participants discussed the relevance of the tool to SBCC programmes. Participants raised several points, including:

- The relevance of the tool and items for SBCC depends on the programme, its process and communication channels.
- Incentives for programmes to use the PRS tool need to be clarified.
- It should also be clarified at what point in the programme process the tool should be used.
- Several supplementary items could be essential – for example, the design process, timing, and provider characteristics
- There are also possible items that could be added, including community ownership and participant voice.

Two participants from the session noted that:

*Taken together, there is a tension between developing a tool that is broad enough to apply to a wide range of fields within the health sector, yet be specific enough so that people feel that it is useful for and applies to their specific topic area.*

*A supplement could be considered for different programme types, i.e. there could be a generic version of the PRS, followed by add-ons and considerations specific to certain disciplines.*

A detailed report of the feedback received during the SBCC session is attached to this background paper.

The draft PRS was also shared with WHO colleagues. It was suggested that the PRS learn from large on-going programmes such as TB, HIV, vaccines, malaria, family planning and child health programmes. They also agreed that Context was an extremely important element and needed to be detailed to ensure important elements were noted. Furthermore, they suggested that the current tool highlights coverage and should dedicate more space to capturing components related to the quality of implementation. Next steps proposed including more in-depth discussion with large donors to determine how this could meet their needs, and testing to determine the usefulness and feasibility of the tool.

**Technical consultation to refine and finalize draft PRS**

Following the Delphi survey, we convened a technical face-to-face consultation to further refine and finalize a draft PRS tool, and to plan for the pilot testing of the tool with SR/MNCAH programmes in different countries globally. Twenty-nine participants attended a meeting on July 12^th^ and 13^th^ 2016 at the WHO headquarters Geneva. Of these, three had participated in the Delphi survey, and six were staff members or consultants at the WHO; 20 had not previously participated in the PRS development. These 20 additional participants were identified to ensure the coverage of the different health areas of SR/MNCAH and different types of health programmes including service delivery, advocacy, social and behavioural interventions, etc.

The meeting had two core objectives:

- To revise and further refine a draft PRS tool developed based on a review of the literature and a Delphi consensus process;
- To develop the methods and plans for further review and pilot testing the PRS with country programmes and experts

Overall, the consultation provided an opportunity to discuss each item in detail, including its consensus as essential or supplementary along with rationales for why, as well as potential ways to edit or merge the item it with other items. Special attention was given to the borderline essential and supplementary items, which required further discussion.

The meeting begun with an overview of the background and development of the PRS tool, followed by a review and discussion of the items; their organization, wording and relevance. The latter session included group work where participants discussed each item in-depth and suggested alternative structures and wordings to the tool. The group work results were presented and discussed during a plenary session on the second day, followed by a consensus discussion about which items should be core, merged, supplementary or excluded. The plenary discussion resulted in an updated list of 28 items across 5 revised domains (Table 5): Programme Overview, Programme Components and Implementation, Monitoring of Implementation, Evaluation and Results, and Synthesis.

The meeting concluded with a discussion about the next steps in the PRS development, including the piloting phase. For starters, the PRS will be piloted through a desk-review with five programmes to assess the relevance and fit of the PRS tool to current program reports. This evaluation will be done in duplicate; i.e. two independent reviewers will review the program reports and assess whether these reports cover the items included in the revised PRS tool. Following the desk review, the PRS will be updated based on the feedback received and the revised version will be described in a WHO guidance document, detailing a first version – PRS 1.0 – for use in the field of SRMNCAH. In addition, results from the Delphi survey, technical consultation and desk review will be disseminated via a peer-reviewed publication.

Following the pilot, the intent is for programmes to use the PRS up-front to structure and guide their reporting. Given that it is the first version, we intend to actively seek feedback on the use of the PRS 1.0 and revisit it in one year’s time following its publication.

**Tables**

**Table 1**. Reporting items related to programme preparation, implementation and evaluation, identified in the Systematic Review

**Table 2**. Characteristics of participants across Delphi rounds

**Table 3.** Summary of Delphi Rounds 1-2 rankings of reporting items, by domain.

**Table 4.** Reporting items for potential inclusion in a PRS tool: Results from Round 3 of the Delphi survey

**Table 5.** DRAFT revised reporting items following the Technical Consultation

**Table 1. Reporting items related to programme preparation, implementation and evaluation, identified in the Systematic Review**

| **Domain** | **Sub-domain** | **Item** | **Description** |
| --- | --- | --- | --- |
| Programme preparation | Objective/Focus | 1. Programme name | Name of programme [4]. |
|  |  | 2. Objectives and anticipated impact of programme (why) | Anticipated short-term and long-term influences of programme on individual participants as well as wider implications [5,6]. |
|  |  | 3. Target population | Characteristics of the target population planned to be reached and at what level (individual, group, wider population) [7-16]. |
|  | Design | 4. Organization/agency | Mention the name, credentials and affiliations of the organization(s) developing the programme [12,17-19]. |
|  |  | 5. Funding source | Name of programme donor/funding source(s) [18-26]. |
|  |  | 6. Programme design process | Description of the process of designing the programme [23,26,27]. |
|  |  | 7. Theoretical foundation | Underlying theory and/or logic model of the programme [4,8,17,27-29], with details for how this theory guided the programme design and messages [30]. |
|  |  | 8. Program manual | Whether a manual or protocol existed for the programme [7,14], and where this can be accessed [31,32]. |
|  |  | 9. Implementation strategy | Details on whether an implementation strategy was developed [6,12,33,34], and if any research questions were specific to implementation [12]. |
|  |  | 10. Evaluation plans | Detail any evaluation plans, both to assess programme implementation/process and to evaluate the programme’s impact/results [4,17,23]. |
|  | Piloting | 11. Piloting of activities | Whether programme activities were piloted, and if so detail how, when, by whom and the results [7,14,17,33,34]. |
| Programme implementation | Content | 12. Components/activities | Define and describe the content of programme activities in enough detail to allow replication [4-10,12,14-16,22,27,29,34-42]. If a control group was used, the content of any activities assigned to the control should also be described [9,27,35,38,40,43]. |
|  |  | 13. Complexity | Degree of complexity of the activities, such as whether single or multiple components were included [5]. |
|  |  | 14. Standardisation | Whether the content of components/activities followed a standardised protocol or curriculum [43]. |
|  |  | 15. Innovation | Degree of innovation as part of the programme [6,33]. |
|  |  | 16. Materials | Type of materials used [4,37,44] and where these can be accessed if applicable [4,8,27]. |
|  | Timing, duration, location | 17. Timing (when) | Timing and duration of the programme (start and finish) [4,5,9,16,29,35,41] . |
|  |  | 18. Setting (where) | Key aspects of the programme setting [4,5,8-10,12,15,27,29,31,32,38,45,46], including geographical context (e.g. country, rural/urban) [47], single/multiple locations [5], type of context [4,15,48] such as “real-world” or clinical [4], and any infrastructure required [4,12]. |
|  |  | 19. Dose and intensity (how much) | Number of sessions/activities, how often activities were delivered [4,8,10,15,35,41], whether the frequency of activities was predetermined or varying [35], and the intensity or duration of each activity [4,9,27,29,35]. |
|  | Providers/staff | 20. Provider characteristics (Who) | Organization(s)/agencies involved in delivering the programme activities [10,15] (name and type) [15], number of staff and their responsibilities [5], staff characteristics including demographics, professions, experience, education and technical skills required [4-6,8-10,27,29,35,43,49-51]. |
|  |  | 21. Provider/staff training | Details on how programme staff was recruited, trained and supervised to deliver activities (when, how and by whom) [7,10,14,35,37]. |
|  |  | 22. Provider reflexivity | Reflection about the relationship between providers and participants, such as whether participants knew the staff [49], influences of professional opinions and the self-efficacy of providers [52]. |
|  | Participants | 23. Participant recruitment | Process of recruiting programme participants [16,35]. |
|  |  | 24. Participants (to whom) | Characteristics of participants that actually received the programme [8,9,27,39,42,48]. Report subgroups by key demographic factors such as age, biological sex/gender, socioeconomic status, education level, religion [7-15,53,54], HIV status, and nr of sexual partners [15]. Note participant risk profiles, if any (e.g. disadvantaged populations) [15,53,54]. |
|  |  | 25. Participant preparation | Whether anything was done to prepare or brief participants prior to the start of the programme [8,37]. |
|  | Delivery | 26. Methods used to deliver activities (how) | Specific methods/channels used for delivering programme activities [4,9,10,15,27,29,35,41,50,51], degrees of human interactivity [5,37,41], level of involvement [48], technology required [10]. |
|  |  | 27. Efforts to ensure fidelity of participants | Efforts to ensure fidelity, increase participation, compliance or adherence, and reduce contamination [4,8,29,35,37,44,52], such as incentives or compensation [4,8,29,35,37,44,52,55]. |
|  |  | 28. Efforts to ensure fidelity of providers/staff | Efforts to enhance adherence of providers [7,10,14,35] such as staff meetings [10], support [7,10], incentives [35,52], feedback [35], motivation [10] and supervision [7]. |
|  | Implementation outcomes | 29. Acceptability | Perception and comfort among stakeholders about the programme, its relative advantage and credibility [12]. |
|  |  | 30. Appropriateness | Perceived fit or relevance of the intervention as judged by the implementers [12]. |
|  |  | 31. Feasibility/practicality | The actual fit, utility or suitability of the programme for the everyday life of participants [12,35]. |
|  |  | 32. Adoption | Uptake/utilization of programme [10,12,35]. Difference in uptake by intervention or control groups, if applicable [35]. |
|  |  | 33. Coverage/Reach | The spread or penetration of the programme components [12,41]. |
|  |  | 34. Attrition | Non-participation and dropout of participants [35], along with reasons for why [35,49]. |
|  |  | 35. Unexpected end of programme | Whether the programme ended or stopped earlier than planned, along with reasons for why [36]. |
|  |  | 36. Reversibility | Whether it would be possible to stop the programme without negative or harmful effects [7,14]. |
|  |  | 37. Contamination of activities | Unanticipated spread of activities outside of the programme target population [10,35]. |
|  |  | 38. Fidelity | Whether the programme was delivered as intended, e.g. discrepancies between the programme design and the actual implementation of components and methods in the "real life context" [4,8,9,12,17,23,27,29,33-35,45,55,56]. |
|  |  | 39. Reasons for low fidelity | Reasons for any deviation from planned activities or others parts of the programme design [4]. |
|  |  | 40. Sustainability | Extent to which participants may be able to use the programme in their everyday life, for example whether any support structures are in place to maintain behaviour changes [35,37], what happened after the program [39], whether any follow-up sessions are planned [37]. |
|  |  | 41. Costs of implementation | Costs and required resources for implementation [7,12,14,33,42], including time, human resources, materials, set-up, administration [7], delivery strategy [12]. |
| Programme evaluation | Process evaluation | 42. Process or implementation evaluation methods | Method that was used to assess implementation outcomes [33,34]. For example, how fidelity was monitored and measured [9]. |
|  |  | 43. Effect of implementation process on results | Whether the implementation process affected results and quality of the programme results [33,34]. |
|  |  | 44. External events affecting implementation | Significant external events occurring at the time of intervention (e.g. social political, economic and/or geographical), which might have affected the implementation [12,17,23,31-33,48,55,56]. |
|  |  | 45. Ethical considerations | Ethical issues that might have affected the implementation [10]. |
|  | Implementation barriers and facilitators | 46. Implementation barriers and facilitators | Detailed description of factors hindering and facilitating implementation of the programme [23,29,33,45,55]. |
|  |  | 47. Strengths and limitations | Appraise weaknesses [17] and strengths [45] in the programme design, what worked and what can be improved [17]. |
|  | Impact/results evaluation | 48. Outcome evaluation methods | How programme results/impact was evaluated [7,14,23,33,34], differentiating between effectiveness, efficacy and cost savings [7]. |
|  |  | 49. Unexpected/negative effects | Any unexpected and/or negative effects of the programme [4,17]. |
|  |  | 50. Differential effects | Whether the programme effects differed according based on characteristics such as biological sex/gender, ethnicity, socioeconomic status, age, geographic location [7,17]. |

| **Table 2.** Characteristics of participants across Delphi rounds | | |  |
| --- | --- | --- | --- |
| \|  \| Round 1 (N=48) \| \| Round 2 (N=29) \| \| Round 3 (N=20) \| \| \| --- \| --- \| --- \| --- \| --- \| --- \| --- \| \|  \| N \| % \| N \| % \| N \| % \| \| **Organization** \|  \|  \|  \|  \|  \|  \| \| UN \| 5 \| 10.4% \| 3 \| 10.7% \| 2 \| 10% \| \| University \| 16 \| 33.3% \| 10 \| 35.7% \| 8 \| 40% \| \| NGO \| 14 \| 29.2% \| 8 \| 28.6% \| 5 \| 25% \| \| Hospital/clinic \| 2 \| 4.2% \| 0 \| 0% \| 0 \| 0% \| \| Donor \| 4 \| 8.3% \| 3 \| 10.7% \| 3 \| 15% \| \| Government \| 5 \| 10.4% \| 3 \| 10.7% \| 1 \| 5% \| \| Other \| 2 \| 4.2% \| 1 \| 3.6% \| 1 \| 5% \| \| **Professional background*** \|  \|  \|  \|  \|  \|  \| \| Programme planner/implementer \| 25 \| 52% \| 13 \| 44.8% \| 9 \| 45% \| \| Researcher/academic \| 31 \| 65.3% \| 15 \| 51.7% \| 11 \| 55% \| \| Medical/clinical \| 16 \| 32.7% \| 8 \| 27.6% \| 7 \| 35% \| \| Management \| 19 \| 37.8% \| 10 \| 34.5% \| 8 \| 40% \| \| Other \| 6 \| 12.2% \| 5 \| 17.2% \| 2 \| 10% \| \| **Region** \|  \|  \|  \|  \|  \|  \| \| Global \| 33 \| 68.8% \| 21 \| 72.4% \| 14 \| 70% \| \| Africa \| 6 \| 12.5% \| 3 \| 10.3% \| 2 \| 10% \| \| Western pacific \| 2 \| 4.2% \| 1 \| 3.4% \| 1 \| 5% \| \| Southeast Asia \| 3 \| 6.3% \| 2 \| 6.9% \| 2 \| 10% \| \| North America \| 3 \| 6.3% \| 2 \| 6.9% \| 1 \| 5% \| \| Europe \| 1 \| 2.1% \| 0 \| 0% \| 0 \| 0% \|   *Multiple response options possible |  |  |  |

**Table 3.** Summary of Delphi Rounds 1-2 rankings of reporting items, by domain.

| **Round 1 ranking (N=43)** | | | | | | **Round 2 ranking (N=29)** | | | | | |
| --- | --- | --- | --- | --- | --- | --- | --- | --- | --- | --- | --- |
|  | **Rating scores** | | **Category of importance**  (% of respondents) | | |  | **Rating scores** | | **Category of importance**  (% of respondents) | | |
| **Original items (systematic review)** | Mean (SD) | Med.  (IQR) | Ess. | Imp. | Not  imp. | **Revised items based on Round 1 feedback** | Mean (SD) | Med. (IQR) | Ess. | Imp. | Not imp. |
| **Programme Preparation** |  |  |  |  |  | **Programme Preparation** |  |  |  |  |  |
| 1. Programme name | 6.9 (2.16) | 7 (5–9) | 58.1 | 37.2 | 4.7 | 1. Programme name | 7.2 (1.7) | 8 (6–9) | 62.1 | 34.5 | 3.5 |
| **2. Overall goal/objectives– anticipated impact** | 8.8 (0.64) | 9 (9–9) | 97.7 | 2.3 | – | **2. Objectives and anticipated effects** | 8.7 (0.7) | 9 (9–9) | 96.6 | 3.5 | – |
| **3. Target population** | 8.4 (1.43) | 9 (8–9) | 93.0 | 4.7 | 2.3 | **3. Target population and area** | 8.5 (0.8) | 9 (8–9) | 96.6 | 3.5 | – |
| 4. Organization/agency | 6.4 (1.97) | 6 (5–8) | 48.8 | 44.2 | 7 | 4. Partners and stakeholder involvement | 7 (1.5) | 7 (6–8) | 65.5 | 34.5 | – |
| 5. Funding source | 6.2 (1.69) | 6 (5–8) | 39.5 | 58.1 | 2.3 | 5. Funding source | 6.4 (1.7) | 6 (5–8) | 44.8 | 48.4 | 6.9 |
| 6. Programme design process | 7.2 (1.63) | 7 (6–9) | 69.8 | 27.9 | 2.3 | 6. Programme design process | 7 (1.5) | 7 (6–8) | 62.1 | 37.9 | – |
| 7. Theoretical foundation | 7.3 (1.84) | 8 (7–9) | 79.1 | 16.3 | 4.7 | **7. Theory and/or logic model** | 7.6 (1.4) | 8 (7–9) | 82.8 | 13.8 | 3.5 |
| 8. Program manual | 6.7 (1.62) | 7 (5–8) | 55.8 | 41.9 | 2.3 | 8. Program manual | 5.8 (1.8) | 6 (5–7) | 41.4 | 48.3 | 10.3 |
| **9. Implementation strategy** | 7.8 (1.36) | 8 (7–9) | 81.4 | 18.6 | – | **9. Implementation strategy** | 7.8 (1.2) | 8 (7–9) | 82.8 | 17.2 | – |
| **10. Evaluation plans** | 8.4 (1.07) | 9 (8–9) | 93.0 | 7.0 | – | **10. Evaluation plans** | 7.9 (1.2) | 8 (7–9) | 85.7 | 14.3 | – |
|  |  |  |  |  | – | 11. Ethical considerations | 7.4 (1.4) | 8 (6–9) | 72.4 | 27.6 | – |
|  |  |  |  |  | – | 12. Dissemination plans | 6.5 (1.9) | 7 (5–8) | 55.2 | 41.4 | 3.5 |
| 11. Piloting of activities |  | 8 (7–8) | 79.1 | 20.9 | – | 13. Piloting of activities | 7 (1.2) | 7 (6–8) | 62.1 | 37.9 | – |
| **Programme Implementation** |  |  |  |  |  | **Programme Implementation** |  |  |  |  |  |
| **12. Components/activities** | 8.4 (1.13) | 9 (8–9) | 88.4 | 11.6 | **–** | **14. Components/activities** | 8.3 (1.2) | 9 (8–9) | 89.7 | 10.3 | – |
| 13. Complexity | 7.2 (1.39) | 7 (6–8) | 66.7 | 33.3 | – | 15. Complexity | 6.8 (1.2) | 7 (6–8) | 37.9 | 62.1 | – |
| **14. Standardisation** | 7.5 (1.32) | 8 (7–9) | 81.4 | 18.6 | **–** | **16. Standardisation and tailoring** | 7.7 (1) | 8 (7–8) | 86.2 | 13.8 | – |
| 15. Innovation | 6.2 (1.75) | 6 (5–8) | 44.2 | 48.8 | 7.0 | *Merged with item #14* | – | – | – | – | – |
| 16. Materials | 7.2 (1.33) | 7 (6–8) | 72.1 | 27.9 | – | 17. Materials | 6.8 (1.6) | 7 (6–8) | 58.6 | 37.9 | 3.5 |
| **17. Timing (when)** | 8.0 (1.37) | 9 (7–9) | 83.3 | 16.7 | **–** | ***18. Timing (when)*** | *7.9 (1.4)* | *9 (7–9)* | *79.3* | *20.7* | *–* |
| **18. Setting (where)** | 8.5 (0.99) | 9 (8–9) | 92.9 | 7.1 | **–** | **19. Setting (where)** | 8.6 (0.8) | 9 (9–9) | 96.6 | 3.5 | – |
| **19. Dose and intensity (how much)** | 8.4 (1.06) | 9 (8–9) | 92.9 | 7.1 | **–** | **20. Dose and intensity (how much)** | 8 (1.2) | 8 (7–9) | 86.2 | 13.8 | – |
| 20. Provider characteristics (by whom) | 7.3 (1.47) | 7.5 (6–9) | 71.4 | 28.6 | – | 21. Provider/staff characteristics (by whom) | 7.3 (1.4) | 7 (6–8) | 72.4 | 27.6 | – |
| 21. Provider/staff training | 7.3 (1.56) | 8 (6–9) | 69.1 | 31 | – | 22. Provider/staff training | 7.1 (1.7) | 7 (6–8) | 65.5 | 31.0 | 3.5 |
| 22. Provider reflexivity | 6.4 (1.94) | 7 (5–8) | 54.8 | 35.7 | 9.5 | 23. Provider reflexivity | 6.2 (1.4) | 6 (5–7) | 46.4 | 53.6 | – |
| 23. Participant recruitment | 7.8 (1.39) | 8 (7–9) | 78.6 | 21.4 | – | 24. Participant recruitment | 7.5 (1.3) | 8 (7–9) | 75.0 | 25.0 | – |
| **24. Participants (who)** | 8.2 (1.13) | 9 (8–9) | 92.9 | 7.1 | **–** | **25. Participants (who)** | 8.2 (1.2) | 9 (8–9) | 86.2 | 13.8 | – |
| 25. Participant preparation | 7.0 (1.64) | 7 (6–8) | 66.7 | 31.0 | 2.3 | 26. Participant preparation (how) | 7.1 (1.4) | 7 (6–8) | 60.7 | 39.3 | – |
| **26. Methods used to deliver activities (how)** | 8.0 (1.21) | 8.5 (7–9) | 85.7 | 14.3 | **–** | **27. Methods used to deliver activities** | 8 (1.2) | 8 (7–9) | 82.8 | 17.2 | – |
| **27. Efforts to ensure fidelity of participants** | 7.7 (1.3) | 8 (7–9) | 82.9 | 17.1 | **–** | ***28. Efforts to increase and sustain participation*** | *7.6 (1.3)* | *8 (7–8)* | *79.3* | *20.7* | *–* |
| **28. Efforts to ensure fidelity of providers/staff** | 7.9 (1.14) | 8 (7–9) | 88.1 | 11.9 | **–** | **29. Efforts to ensure provider adherence to protocol** | 7.7 (1.2) | 8 (7–9) | 85.7 | 14.3 | – |
|  |  |  |  |  | – | **30. Monitoring of the programme implementation** | 7.6 (1.2) | 8 (7–9) | 82.1 | 17.9 | **–** |
| **29. Acceptability** | 7.9 (1.13) | 8 (7–9) | 88.1 | 11.9 | **–** | 31. Acceptability | 7.3 (1.4) | 8 (6–8) | 72.4 | 27.6 | – |
| **30. Appropriateness** | 7.7 (1.2) | 8 (7–9) | 85.7 | 14.3 | **–** | 32. Appropriateness | 7.2 (1.2) | 7 (6–8) | 69.0 | 31.0 | – |
| **31. Feasibility/practicality** | 8.0 (1.08) | 8 (8–9) | 90.5 | 9.5 | **–** | **33. Feasibility/practicality** | 7.9 (1) | 8 (7–9) | 89.7 | 10.3 | **–** |
| **32. Adoption** | 8.5 (0.97) | 9 (8–9) | 92.9 | 7.1 | **–** | **34. Adoption** | 8.2 (0.9) | 8 (8–9) | 96.6 | 3.5 | **–** |
| **33. Coverage/Reach** | 8.1 (1.13) | 8.5 (8–9) | 90.5 | 9.5 | **–** | **35. Coverage/Reach** | 8 (1.1) | 8 (8–9) | 93.1 | 6.9 | **–** |
| **34. Attrition** | 8.1 (1.09) | 8 (8–9) | 88.1 | 11.9 | **–** | **36. Attrition** | 7.7 (1.1) | 8 (7–9) | 82.8 | 17.2 | **–** |
| **35. Unexpected end of programme** | 8.5 (0.83) | 9 (8–9) | 97.6 | 2.4 | **–** | **37. Unexpected end of programme** | 8.1 (1.2) | 8.5 (8–9) | 89.3 | 10.7 | **–** |
| 36. Reversibility | 7.1 (1.26) | 7 (6–8) | 65.8 | 34.2 | – | 38. Reversibility | 6.5 (1.3) | 6 (6–8) | 46.2 | 53.9 | – |
| 37. Contamination of activities | 7.3 (1.44) | 8 (6–8) | 69.1 | 30.9 | – | 39. Contamination of activities | 7.3 (1) | 7 (6–8) | 74.1 | 25.9 | – |
| **38. Fidelity** | 8.2 (1.03) | 8 (8–9) | 90.2 | 9.8 | **–** | **40. Fidelity** | 8.4 (0.9) | 9 (8–9) | 92.9 | 7.1 | **–** |
| **39. Reasons for low fidelity** | 7.9 (1.09) | 8 (8–9) | 88.1 | 11.9 | **–** | **41. Reasons for low fidelity** | 7.9 (1.1) | 8 (7–9) | 85.2 | 14.8 | **–** |
| **40. Sustainability** | 8.3 (1.09) | 9 (8–9) | 88.1 | 11.9 | **–** | *Moved to item #49* |  |  |  |  | – |
| **41. Costs of implementation** | 8.2 (0.98) | 9 (8–9) | 92.9 | 7.1 | **–** | **42. Costs of implementation** | 8.1 (1.1) | 8 (7–9) | 93.1 | 6.9 | – |
| **Programme Evaluation** |  |  |  |  |  | **Programme Evaluation** |  |  |  |  |  |
| **42. Process evaluation methods** | 8.5 (0.95) | 9 (8–9) | 95.1 | 4.9 | – | **43. Process evaluation methods** | 8.3 (1) | 9 (8–9) | 89.7 | 10.3 | – |
| **43. Effect of implementation process on results** | 8.3 (0.97) | 9 (8–9) | 95.0 | 5.0 | – | **44. Effect of implementation process on results** | 8.1 (1) | 8 (8–9) | 89.7 | 10.3 | – |
| **44. External events affecting implementation** | 8.1 (0.9) | 8 (8–9) | 95.1 | 4.9 | – | **45. Factors affecting implementation** | 8.2 (0.9) | 8 (8–9) | 93.1 | 6.9 | – |
| **45. Ethical considerations** | 8.2 (0.88) | 8 (8–9) | 95.1 | 4.9 | – | *Moved to item #11* | – | – | – | – | – |
| **46. Implementation barriers and facilitators** | 8.1 (0.93) | 8 (8–9) | 97.6 | 2.4 | – | *Moved to item #50* | – | – | – | – | – |
| **47. Strengths and limitations** | 8.2 (0.87) | 8 (8–9) | 95.2 | 4.8 | – | *Moved to item #50* | – | – | – | – | – |
| **48. Outcome evaluation methods** | 8.5 (1.17) | 9 (8–9) | 90.5 | 9.5 | – | **46. Outcome evaluation methods** | 8.4 (0.8) | 9 (8–9) | 96.6 | 3.5 | – |
| **49. Unexpected/negative effects** | 8.3 (0.82) | 9 (8–9) | 97.6 | 2.4 | – | **47. Unexpected programme effects** | 8.2 (0.9) | 8 (8–9) | 93.1 | 6.9 | – |
| **50. Differential effects** | 8.0 (1.09) | 8 (7–9) | 88.1 | 11.9 | – | **48. Differential effects** | 8.2 (1) | 8 (8–9) | 89.7 | 10.3 | – |
|  |  |  |  |  |  | **49. Sustainability** | 7.8 (0.9) | 8 (7–8) | 92.6 | 7.4 | – |
|  |  |  |  |  |  | **50. Strengths and limitations (lessons learnt)** | 8.4 (0.9) | 9 (8–9) | 96.4 | 3.6 | – |
|  |  |  |  |  |  |  |  |  |  |  |  |

**Table 4.** Reporting items for potential inclusion in a PRS tool: Results from Round 3 of the Delphi survey

| **A. Programme Preparation** | | | |  |
| --- | --- | --- | --- | --- |
| **Sub-domain** | **Item** | **Item description** | **Item ranking** | **Consensus**  **(% agree)*** |
| A1. Objectives/  Focus | 1. Programme name | Name of programme. | Supplementary |  |
|  | 1. **Objectives and anticipated effects** | Overall programme goal and objectives based on clearly defined criteria. Anticipated short-term and long-term influences of programme at different levels (e.g. participants, communities, society). | Essential | 100% |
|  | 1. **Target population and area** | Characteristics of the target population to be reached and at what level (e.g. individual, group, community), including planned geographical area(s) of scale. | Essential | 95% |
| A2. Design | 1. Partners and stakeholder involvement | Who was involved in developing the programme (organization/partners); their role, including stakeholder consultations and local buy-in from community members. | Supplementary |  |
|  | 1. Funding source | Name of programme donor/funding source(s). | Supplementary |  |
|  | 1. Programme design process | Detail how and why activities were decided upon (e.g. background/rationale, current gaps in programming, risks and benefits, and the evidence-base (if any) of activities. | Supplementary |  |
|  | 1. ***Theory and/or logic model*** | Theoretical foundation and/or logic model framework underlying the programme, with details for how this guided programme design. | Borderline essential | 75% |
|  | 1. Program manual | Whether a manual or protocol existed for the programme, and where this can be accessed. | Supplementary |  |
|  | 1. **Implementation strategy** | Details on whether an implementation strategy was developed, including the planned programme activities and a description of the implementation timeline. | Essential | 85% |
|  | 1. **Evaluation plans** | Any evaluation plans, both to assess the programme implementation process and to evaluate results, including up-front definition of indicators. | Essential | 85% |
|  | 1. Ethical considerations | Ethical considerations of the programme, including details on the accountability framework (if applicable). | Supplementary |  |
|  | 1. Dissemination plans | Plans for disseminating information about how the programme (e.g. its design and implementation, in a publicly accessible report or article. | Supplementary |  |
| A3. Piloting | 1. Piloting of activities | Whether programme activities were piloted; if so detail how, when, where, by whom and the results. | Supplementary |  |
| **B. Programme Implementation** | | | |  |
| **Sub-domain** | **Item** | **Item description** | **Item ranking** | **Consensus**  **(% agree)*** |
| B1. Content | 1. **Components/**   **activities** | Describe the core components/activities of the programme in enough detail to allow replication. This may include the complexity and innovation of activities. Include control group activities, if applicable. | Essential | 100% |
|  | 1. **Standardisation and tailoring** | Whether the content and implementation of activities followed a standardised protocol; may incorporate tailoring of the activities (e.g. to different subgroups or contexts). | Essential | 90% |
|  | 1. Materials | Type of materials used and where these can be accessed (if applicable). | Supplementary |  |
| B2. Timing, duration, location | 1. ***Timing (when)*** | Timing and duration of the programme (start and finish). | Borderline essential | 65% |
|  | 1. **Setting (where)** | Key aspects of the programme setting, such as the type of context (e.g. geographical, political, clinical) and number of locations. | Essential | 90% |
|  | 1. **Dose and intensity (how much)** | Number of sessions/activities; how often and for how long activities were delivered, whether the frequency of activities was predetermined or varying. | Essential | 85% |
| B3. Programme providers/staff | 1. Provider/staff characteristics (by whom) | Organization(s) involved in delivering the programme, staff responsibilities and characteristics (e.g. skills and experience) and community participation in programme delivery. | Supplementary |  |
|  | 1. Provider/staff training | Details on how programme staff was recruited, trained and supervised to deliver activities (when, how and by whom). | Supplementary |  |
|  | 1. Provider reflexivity | Reflection about the relationship between provides and participants, e.g. influences of professional opinions and the self-efficacy of providers. | Supplementary |  |
| B4. Programme participants | 1. Participant recruitment | Process of recruiting programme participants. | Supplementary |  |
|  | 1. **Participants (who)** | Characteristics of participants that actually received the programme, including key demographic factors such as age, gender, SES, education level, religion. Note any risk profile that is relevant to the programme. | Essential | 90% |
|  | 1. Participant preparation | Whether anything was done to prepare or brief participants about the programme prior to the start of activities. | Supplementary |  |
| B5. Delivery | 1. **Methods used to deliver activities (how)** | Detail the specific methods that were used for delivering each programme activity. | Essential | 90% |
|  | 1. ***Efforts to increase and sustain participation*** | Efforts to increase and sustain participation and adherence, e.g. incentives, community sensitization and involvement. | Borderline essential | 70% |
|  | 1. **Efforts to ensure provider adherence to protocol** | Efforts to enhance adherence of providers/staff to study protocol, e.g. staff meetings, incentives, feedback. | Essential | 85% |
|  | 1. **Monitoring of the programme implementation** | Daily/regular monitoring of the implementation process including the collection and analysis of indicators, identification and timely resolution of problems. | Essential | 100% |
| B6. Implementation outcomes | 1. Acceptability | The extent to which the programme is considered to be reasonable among stakeholders (i.e. those receiving, delivering or otherwise affected by it). | Supplementary |  |
|  | 1. Appropriateness | Perceived fit or relevance of the intervention as judged by the implementers. | Supplementary |  |
|  | 1. **Feasibility/**   **practicality** | The feasibility of delivering activities as well as their actual fit or suitability for the everyday life of participants. | Essential | 90% |
|  | 1. **Adoption** | Uptake/utilization of programme. Difference in uptake by intervention or control groups, if applicable. | Essential | 95% |
|  | 1. **Coverage/Reach** | The coverage (spread) of the programme components, including details on differential reach of different programme components, including outside of the target population. | Essential | 95% |
|  | 1. **Attrition** | Non-participation and dropout of participants, along with reasons for why. | Essential | 95% |
|  | 1. **Unexpected end of programme** | Whether the programme ended or stopped earlier than planned, along with reasons for why. | Essential | 85% |
|  | 1. Reversibility | Whether it would be possible to stop the programme without negative or harmful effects. | Supplementary |  |
|  | 1. **Fidelity** | Whether the programme was delivered as intended, e.g. discrepancies between the programme design and the actual implementation of components and methods. Note reasons for low fidelity, if applicable. | Essential | 85% |
|  | 1. **Implementation costs/resources** | Required resources for implementation (e.g. time, human resources, materials, set-up, administration), including the costs associated with these resources where available. | Essential | 80% |
| **C. Programme Evaluation** | | | |  |
| **Sub-domain** | **Item** | **Item description** | **Item ranking** | **Consensus**  **(% agree)*** |
| C1. Implementation evaluation process | 1. **Process evaluation methods** | How, when and by who implementation processes and outcomes (e.g. fidelity) were evaluated. | Essential | 100% |
|  | 1. **Effect of implementation process on results** | Whether the implementation process affected programme results and its quality. | Essential | 100% |
|  | 1. **Factors affecting implementation** | Detailed description of factors hindering and facilitating programme implementation. May include external events (e.g. social, political, economic and/or geographical). | Essential | 100% |
| Outcome/results evaluation process | 1. **Outcome evaluation methods** | How, when and by who programme outcomes/impact was evaluated. | Essential | 95% |
|  | 1. **Unexpected programme effects** | Whether the programme had an effect on participants (whether positive or negative), their lives or communities, beyond what was anticipated in the design. | Essential | 90% |
|  | 1. **Differential effects** | Whether the programme effects differed based on characteristics such as gender, ethnicity, SES, age, geographic location. | Essential | 90% |
|  | 1. **Sustainability** | Extent to which participants may be able to use the programme in their everyday life, e.g. support structures to maintain behaviour changes, what happened after the program, planned follow-up sessions. | Essential | 80% |
|  | 1. **Strengths and limitations (lessons learnt)** | Appraise weaknesses and strengths in the programme design, what worked and what can be improved (i.e. lessons learnt). | Essential | 90% |

* Note: Bolded items were ranked as **essential** for a PRS tool by at least 80% of participants in round 3 of the Delphi survey, while items below this cut-point could be considered as supplementary items. Items ranked as essential by 70-79% of participants are marked as ***borderline essential.***

**Table 5.** DRAFT Updated list of PRS items following the Technical Consultation on July 12-13, 2016.

| **A. Programme Overview**  This section provides an overview of the programme; its background, objectives, start- and end date, where it took place, stakeholders, underlying theory of change, and human rights considerations. | | | |
| --- | --- | --- | --- |
| **Nr** | **Topic** | **Item and description** | **Reported in (source and page)** |
| 1 | Rationale, objectives and anticipated effects | 1a. Programme rationale, i.e. why was the programme conducted. |  |
|  |  | 1b. Goals and objectives. |  |
|  |  | 1c. Anticipated short- and long-term effects of programme at different levels (e.g. individual, organization, community, society). |  |
| 2 | Start and end date | 2a. Planned start- and end date of the programme. |  |
|  |  | 2b. Delays and/or unexpected end of the programme along with reasons why (if applicable). |  |
| 3 | Setting and Context | 3a. Where the programme took place, e.g. country name(s), specific locations, urban/rural environments. |  |
|  |  | 3b. Overview of the context (e.g. political, historical, social, cultural, health system) pertinent to the programme. |  |
| 4 | Stakeholders | 4a. Programme target population (key sociodemographic characteristics such as age, gender, ethnicity, education level) |  |
|  |  | 4b. Implementing organisation(s). |  |
|  |  | 4c. Partners and other stakeholders (e.g. community leaders). |  |
|  |  | 4d. How the different stakeholders (4a-c) were involved in programme development and/or implementation. |  |
| 5 | Funding source(s) | 5. Name of programme donor/funding source(s). |  |
| 6 | Theory of change and/or logic model | 6. Theory of change, assumptions, and/or logic model framework underlying the programme, with details for how this guided the programme design, implementation and evaluation plans. |  |
| 7 | Human rights perspectives | 7a. If and how gender, equity, rights and ethical considerations were integrated into the programme. |  |
|  |  | 7b. Accountability framework is adapted that defines the programme’s commitments and how it will be accountable for these commitments (if applicable). |  |
| **B. Programme Components and Implementation**  This section relates to the programme components/activities, including the development and piloting of activities; a description of the core components and how these were implemented (what, how, when, by who, and for whom); and quality assurance mechanisms. | | | |
| **Nr** | **Topic** | **Item and description** | **Reported in (source and page)** |
| 8 | Programme planning | 8. How and why activities were decided upon. May include situational analysis, current gaps and needs in programming, the evidence-base of activities, planned adaptation of activities, risks and benefits, plans for scalability and/or sustainability). |  |
| 9 | Piloting | 9. Piloting of the programme activities elsewhere or within the programme , and if so how, when, where, by whom and with what results. |  |
| 10 | Components/Activities (Please repeat for each component) | 10. Detailed description of the core programme components/activities, including:   - **What** was done - **How** (implementation methods/delivery processes/approaches). - **When** (frequency, intensity, duration). - **By whom** (characteristics, skills, training and responsibilities of implementing personnel (i.e. staff, providers, volunteers). - **For whom** (target population for each activity). - **Support materials** used and where these can be accessed (if applicable).   Highlight the innovation of activities, if applicable. |  |
| 11 | Quality assurance mechanisms | 11a. Mechanisms used to ensure the quality in the delivery of activities (e.g. supervision and support of implementing personnel, refresher trainings, product quality checks). |  |
|  |  | 11b. Efforts used to increase and sustain participation and adherence, e.g. community involvement, incentives. |  |
| **C. Monitoring of Implementation**  This section describes the programme monitoring process, and key implementation indicators including the programme’s coverage/reach, fidelity and adaption, acceptability, feasibility, and overall factors affecting the implementation. | | | |
| **Nr** | **Topic** | **Item and description** | **Reported in (source and page)** |
| 12 | Monitoring mechanisms | 12. How the programme implementation process was monitored, including the collection and analysis of indicators used for identifying problems. |  |
| 13 | Coverage/Reach and Drop-out | 13a. Differential uptake (utilization) each programme activity reported by key sociodemographics. |  |
|  |  | 13b. Coverage (spread) of the programme activities, including differential reach in or outside of the target population. |  |
|  |  | 13c. Non-participation and dropout among the target population, along with key sociodemographics and reasons for why. |  |
| 14 | Adaptation | 14a. Whether the programme was delivered as intended, e.g. discrepancies between programme design vs. the actual implementation of components, degree of match between programme content and theory of change. |  |
|  |  | 14b. On-going adaptation of the programme activities to better fit the context, and the fidelity to the adapted activity plan. |  |
| 15 | Acceptability | 15. Acceptability of the programme among stakeholders, i.e., assessment of whether the programme was considered to be reasonable and relevant. |  |
| 16 | Feasibility | 16. Assessment of the feasibility of activities in terms of delivery and actual fit or suitability for participants |  |
| 17 | Factors affecting implementation | 17. Description of barriers to and facilitators of programme implementation. May include contextual factors (e.g. social, political, economic, health systems). |  |
| **D. Evaluation and Results**  This section provides an overview of how the programme was evaluated along with key results, differential and unexpected effects, and costs. | | | |
| **Nr** | **Topic** | **Item and description** | **Reported in (source and page)** |
| 18 | Evaluation | 18a. Type of evaluation(s) conducted (e.g. process evaluation and/or outcome evaluation, quantitative or qualitative). |  |
|  |  | 18b. Evaluation methods. How, when (timing and phases e.g. baseline, midline, end line) and by who the programme was evaluated.^[[1]](#footnote-1)^ |  |
| 19 | Results | 19a. Description of the programme evaluation results (process and/or outcome), differentiating between short/mid/long-term effects. |  |
|  |  | 19b. Whether the programme effects differed across key sociodemographic characteristics and/or geographical areas. |  |
|  |  | 19c. Whether the programme had unexpected effects (beyond what was anticipated in the design) on the target population, health services and/or the communities, |  |
| 20 | Costs | 20a. Summary of the required resources for implementation (e.g., financial, time, human resources, materials, administration) |  |
|  |  | 20b. If and how a cost analysis or cost-effectiveness analysis was conducted, along with results (if applicable). |  |
| **E. Synthesis**  This section provides a synthesis of reflections on the scalability and sustainability of the programme, possibilities for adaptation by other organizations and in other settings, and overall lessons learnt. | | | |
| **Nr** | **Topic** | **Item and description** | **Reported in (source and page)** |
| 21 | Lessons learnt | 21. Appraised weaknesses and strengths of the programme, what worked and what can be improved. |  |
| 22 | Sustainability | 22. Reflections on the sustainability of the programme, i.e. the expected ability to maintain the programme activities (e.g. follow-up sessions), engagement, effects (e.g. behavioural changes) and/or partnerships over time. |  |
| 23 | Scalability | 23. Description of the scale-up of all or some programme activities, or any plans for scale-up. |  |
| 24 | Possibilities for implementation in other settings | 24. Reflections on the context-dependence of the programme and (and with what degree of effort) it could be implemented in/adapted to other settings. |  |
| 25 | Additional information (optional) | References and/or links to additional sources of information in relation to the programme. |  |

**References**

1. Moher D, Schulz KF, Simera I, Altman DG. Guidance for developers of health research reporting guidelines. PLoS Med. 2010;7: e1000217.

2. Kågesten A, Tunçalp Ö, Ali M, Chandra-Mouli V, Tran N, Gulmezoglu A. A Systematic Review of Reporting Tools applicable to Sexual and Reproductive Health Programmes: Step 1 in Developing Programme Reporting Standards. Forthcoming in PLOS One. 2015.

3. Hasson F, Keeney S, McKenna H. Research guidelines for the Delphi survey technique. Journal of advanced nursing. 2000;32: 1008-1015.

4. Hoffmann TC, Glasziou PP, Boutron I, Milne R, Perera R, Moher D, et al. Better reporting of interventions: Template for intervention description and replication (TIDieR) checklist and guide. BMJ (Online). 2014;348.

5. Wells M, Williams B, Treweek S, Coyle J, Taylor J. Intervention description is not enough: evidence from an in-depth multiple case study on the untold role and impact of context in randomised controlled trials of seven complex interventions. Trials. 2012;13: 95.

6. Flores SA, Crepaz N. Quality of study methods in individual- and group-level HIV intervention research: critical reporting elements. AIDS Educ Prev. 2004;16: 341-352.

7. Bird VJ, Le Boutillier C, Leamy M, Williams J, Bradstreet S, Slade M. Evaluating the feasibility of complex interventions in mental health services: Standardised measure and reporting guidelines. British Journal of Psychiatry. 2014;204: 316-321.

8. Conn VS, Groves PS. Protecting the power of interventions through proper reporting. Nurs Outlook. 2011;59: 318-325.

9. Davidson KW, Goldstein M, Kaplan RM, Kaufmann PG, Knatterud GL, Orleans CT, et al. Evidence-based behavioral medicine: what is it and how do we achieve it? Ann Behav Med. 2003;26: 161-171.

10. Montgomery P, Underhill K, Gardner F, Operario D, Mayo-Wilson E. The Oxford Implementation Index: a new tool for incorporating implementation data into systematic reviews and meta-analyses. J Clin Epidemiol. 2013;66: 874-882.

11. O'Neill J, Tabish H, Welch V, Petticrew M, Pottie K, Clarke M, et al. Applying an equity lens to interventions: using PROGRESS ensures consideration of socially stratifying factors to illuminate inequities in health. J Clin Epidemiol. 2014;67: 56-64.

12. Peters DH, Adam T, Alonge O, Agyepong IA, Tran N. Implementation research: what it is and how to do it. Bmj. 2013;347.

13. Kavanagh J, S O, Lorenc (2008) Reflections in developing and using. PROGRESS-Plus. . Equity Update.

14. Chalmers I, Bracken MB, Djulbegovic B, Garattini S, Grant J, Gülmezoglu AM, et al. How to increase value and reduce waste when research priorities are set. The Lancet383: 156-165.

15. Thomas CW, Smith BD, Wright-DeAgüero L. The Program Evaluation and Monitoring System: A Key Source of Data for Monitoring Evidence-Based HIV Prevention Program Processes and Outcomes. AIDS Education and Prevention. 2006;18: 74-80.

16. Bennett C, Khangura S, Brehaut JC, Graham ID, Moher D, Potter BK, et al. Reporting guidelines for survey research: an analysis of published guidance and reporting practices. PLoS Med. 2010;8: e1001069.

17. Armstrong R, Waters E, Moore L, Riggs E, Cuervo LG, Lumbiganon P, et al. Improving the reporting of public health intervention research: Advancing TREND and CONSORT. Journal of Public Health. 2008;30: 103-109.

18. Eysenbach G. CONSORT-EHEALTH: Improving and standardizing evaluation reports of web-based and mobile health interventions. Journal of Medical Internet Research. 2011;13: 25-34.

19. Baker TB, Gustafson DH, Shaw B, Hawkins R, Pingree S, Roberts L, et al. Relevance of CONSORT reporting criteria for research on eHealth interventions. Patient Educ Couns. 2010;81 Suppl: S77-86.

20. von Elm E, Altman DG, Egger M, Pocock SJ, Gotzsche PC, Vandenbroucke JP. The Strengthening the Reporting of Observational Studies in Epidemiology (STROBE) statement: guidelines for reporting observational studies. PLoS Med. 2007;4: e296.

21. Vandenbroucke JP, von Elm E, Altman DG, Gotzsche PC, Mulrow CD, Pocock SJ, et al. Strengthening the Reporting of Observational Studies in Epidemiology (STROBE): explanation and elaboration. PLoS Med. 2007;4: e297.

22. Altman DG, Schulz KF, Moher D, Egger M, Davidoff F, Elbourne D, et al. The revised CONSORT statement for reporting randomized trials: explanation and elaboration. Ann Intern Med. 2001;134: 663-694.

23. Ogrinc G, Mooney SE, Estrada C, Foster T, Goldmann D, Hall LW, et al. The SQUIRE (Standards for QUality Improvement Reporting Excellence) guidelines for quality improvement reporting: explanation and elaboration. Qual Saf Health Care. 2008;17 Suppl 1: i13-32.

24. Husereau D, Drummond M, Petrou S, Carswell C, Moher D, Greenberg D, et al. Consolidated Health Economic Evaluation Reporting Standards (CHEERS) statement. Value Health. 2013;16: e1-5.

25. Husereau D, Drummond M, Petrou S, Carswell C, Moher D, Greenberg D, et al. Consolidated Health Economic Evaluation Reporting Standards (CHEERS)--explanation and elaboration: a report of the ISPOR Health Economic Evaluation Publication Guidelines Good Reporting Practices Task Force. Value Health. 2013;16: 231-250.

26. Davidoff F, Batalden P, Stevens D, Ogrinc G, Mooney S. Publication guidelines for quality improvement in health care: evolution of the SQUIRE project. Qual Saf Health Care. 2008;17 Suppl 1: i3-9.

27. Albrecht L, Archibald M, Arseneau D, Scott SD. Development of a checklist to assess the quality of reporting of knowledge translation interventions using the Workgroup for Intervention Development and Evaluation Research (WIDER) recommendations. Implement Sci. 2013;8: 52.

28. Abraham C, Johnson BT, de Bruin M, Luszczynska A. Enhancing reporting of behavior change intervention evaluations. J Acquir Immune Defic Syndr. 2014;66 Suppl 3: S293-299.

29. Des Jarlais DC, Lyles C, Crepaz N. Improving the reporting quality of nonrandomized evaluations of behavioral and public health interventions: the TREND statement. Am J Public Health. 2004;94: 361-366.

30. Harrington NG, Noar SM. Reporting standards for studies of tailored interventions. Health Education Research. 2012;27: 331-342.

31. Talmon J, Ammenwerth E, Brender J, de Keizer N, Nykanen P, Rigby M. STARE-HI--Statement on reporting of evaluation studies in Health Informatics. Int J Med Inform. 2009;78: 1-9.

32. Rigby M, Talmon J, Brender J, Ammenwerth E, De Keizer NF, Nykanen P. Linking informaticians and end users - Using the STARE-HI evaluation reporting framework as a unifying design approach. In: Adlassnig KP, editor. Medical Informations in a United and Healthy Europe: 2009 European Federation for Medical Informatics; 2009: pp. 66-70.

33. Möhler R, Bartoszek G, Köpke S, Meyer G. Proposed criteria for reporting the development and evaluation of complex interventions in healthcare (CReDECI ): Guideline development. International Journal of Nursing Studies. 2012;49: 40-46.

34. Mohler R, Bartoszek G, Meyer G. Quality of reporting of complex healthcare interventions and applicability of the CReDECI list - a survey of publications indexed in PubMed. BMC medical research methodology. 2013;13: 125.

35. Mayo-Wilson E, Grant S, Hopewell S, Macdonald G, Moher D, Montgomery P. Developing a reporting guideline for social and psychological intervention trials. Trials. 2013;14: 242.

36. Moher D, Hopewell S, Schulz KF, Montori V, Gotzsche PC, Devereaux PJ, et al. CONSORT 2010 Explanation and Elaboration: Updated guidelines for reporting parallel group randomised trials. J Clin Epidemiol. 2010;63: e1-37.

37. Phillips AC, Lewis LK, McEvoy MP, Galipeau J, Glasziou P, Hammick M, et al. A Delphi survey to determine how educational interventions for evidence-based practice should be reported: stage 2 of the development of a reporting guideline. BMC Med Educ. 2014;14: 159.

38. Zwarenstein M, Treweek S, Gagnier JJ, Altman DG, Tunis S, Haynes B, et al. Improving the reporting of pragmatic trials: an extension of the CONSORT statement. Bmj. 2008;337: a2390.

39. Fernald D, Harris A, Deaton EA, Weister V, Pray S, Baumann C, et al. A standardized reporting system for assessment of diverse public health programs. Preventing Chronic Disease. 2012;9.

40. Liberati A, Altman DG, Tetzlaff J, Mulrow C, Gotzsche PC, Ioannidis JP, et al. The PRISMA statement for reporting systematic reviews and meta-analyses of studies that evaluate health care interventions: explanation and elaboration. PLoS Med. 2009;6: e1000100.

41. Proudfoot J, Klein B, Barak A, Carlbring P, Cuijpers P, Lange A, et al. Establishing guidelines for executing and reporting internet intervention research. Cognitive Behaviour Therapy. 2011;40: 82-97.

42. Ramsey S, Willke R, Briggs A, Brown R, Buxton M, Chawla A, et al. Good Research Practices for Cost-Effectiveness Analysis Alongside Clinical Trials: The ISPOR RCT-CEA Task Force Report. Value in Health. 2005;8: 521-533.

43. Boutron I, Moher D, Tugwell P, Giraudeau B, Poiraudeau S, Nizard R, et al. A checklist to evaluate a report of a nonpharmacological trial (CLEAR NPT) was developed using consensus. J Clin Epidemiol. 2005;58: 1233-1240.

44. Phillips AC, Lewis LK, McEvoy MP, Galipeau J, Glasziou P, Hammick M, et al. A systematic review of how studies describe educational interventions for evidence-based practice: Stage 1 of the development of a reporting guideline. BMC Medical Education. 2014;14.

45. Roen K, Arai L, Roberts H, Popay J. Extending systematic reviews to include evidence on implementation: methodological work on a review of community-based initiatives to prevent injuries. Soc Sci Med. 2006;63: 1060-1071.

46. MacPherson H, Jobst KA. Improving the Reporting of Interventions in Clinical Trials of Acupuncture: The Updated and Revised STRICTA. Journal of Alternative and Complementary Medicine. 2010;16: 929-930.

47. Matsumoto M, Bowman R, Worley P. A guide to reporting studies in rural and remote health. Rural Remote Health. 2012;12: 2312.

48. Staniszewska S, Brett J, Mockford C, Barber R. The GRIPP checklist: strengthening the quality of patient and public involvement reporting in research. Int J Technol Assess Health Care. 2011;27: 391-399.

49. Tong A, Sainsbury P, Craig J. Consolidated criteria for reporting qualitative research (COREQ): A 32-item checklist for interviews and focus groups. International Journal for Quality in Health Care. 2007;19: 349-357.

50. Bossuyt PM, Reitsma JB, Bruns DE, Gatsonis CA, Glasziou PP, Irwig LM, et al. The STARD statement for reporting studies of diagnostic accuracy: explanation and elaboration. Ann Intern Med. 2003;138: W1-12.

51. Bossuyt PM, Reitsma JB, Bruns DE, Gatsonis CA, Glasziou PP, Irwig LM, et al. Towards complete and accurate reporting of studies of diagnostic accuracy: The STARD Initiative. Ann Intern Med. 2003;138: 40-44.

52. Flottorp SA, Oxman AD, Krause J, Musila NR, Wensing M, Godycki-Cwirko M, et al. A checklist for identifying determinants of practice: a systematic review and synthesis of frameworks and taxonomies of factors that prevent or enable improvements in healthcare professional practice. Implement Sci. 2013;8: 35.

53. Welch V, Petticrew M, Tugwell P, Moher D, O'Neill J, Waters E, et al. PRISMA-Equity 2012 Extension: Reporting Guidelines for Systematic Reviews with a Focus on Health Equity. PLoS Medicine. 2012;9.

54. Burford BJ, Welch V, Waters E, Tugwell P, Moher D, O'Neill J, et al. Testing the PRISMA-Equity 2012 Reporting Guideline: The Perspectives of Systematic Review Authors. PLoS ONE. 2013;8.

55. Mayo-Wilson E. Reporting implementation in randomized trials: proposed additions to the consolidated standards of reporting trials statement. Am J Public Health. 2007;97: 630-633.

56. Montgomery P, Grant S, Hopewell S, Macdonald G, Moher D, Michie S, et al. Protocol for CONSORT-SPI: An extension for social and psychological interventions. Implementation Science. 2013;8.

1. Reports of research studies should provide further details in line with guidelines for the reporting of the specific study design. Different guidelines are available in the EQUATOR database (<http://www.equator-network.org/>). [↑](#footnote-ref-1)
